# Early Predictors of Impaired Social Functioning in Male Rhesus Macaques (*Macaca mulatta*)

**DOI:** 10.1371/journal.pone.0165401

**Published:** 2016-10-27

**Authors:** Valentina Sclafani, Laura A. Del Rosso, Shannon K. Seil, Laura A. Calonder, Jesus E. Madrid, Kyle J. Bone, Elliott H. Sherr, Joseph P. Garner, John P. Capitanio, Karen J. Parker

**Affiliations:** 1 Winnicott Research Unit, University of Reading, RG6 6AL, Reading, United Kingdom; 2 California National Primate Research Center, University of California Davis, Davis, California, 95616, United States of America; 3 Neurosciences Program, Stanford University, Stanford, California, 94305, United States of America; 4 Department of Psychiatry and Behavioral Sciences, Stanford University, Stanford, California, 94305, United States of America; 5 Department of Neurology, University of California San Francisco, San Francisco, California, 94143, United States of America; 6 Department of Comparative Medicine, Stanford University, Stanford, California, 94305, United States of America; 7 Department of Psychology, University of California Davis, Davis, California, 95616, United States of America; University of Portsmouth, UNITED KINGDOM

## Abstract

Autism spectrum disorder (ASD) is characterized by social cognition impairments but its basic disease mechanisms remain poorly understood. Progress has been impeded by the absence of animal models that manifest behavioral phenotypes relevant to ASD. Rhesus monkeys are an ideal model organism to address this barrier to progress. Like humans, rhesus monkeys are highly social, possess complex social cognition abilities, and exhibit pronounced individual differences in social functioning. Moreover, we have previously shown that Low-Social (LS) vs. High-Social (HS) adult male monkeys exhibit lower social motivation and poorer social skills. It is not known, however, when these social deficits first emerge. The goals of this study were to test whether juvenile LS and HS monkeys differed as infants in their ability to process social information, and whether infant social abilities predicted later social classification (i.e., LS vs. HS), in order to facilitate earlier identification of monkeys at risk for poor social outcomes. Social classification was determined for N = 25 LS and N = 25 HS male monkeys that were 1–4 years of age. As part of a colony-wide assessment, these monkeys had previously undergone, as infants, tests of face recognition memory and the ability to respond appropriately to conspecific social signals. Monkeys later identified as LS vs. HS showed impairments in recognizing familiar vs. novel faces and in the species-typical adaptive ability to gaze avert to scenes of conspecific aggression. Additionally, multivariate logistic regression using infant social ability measures perfectly predicted later social classification of all N = 50 monkeys. These findings suggest that an early capacity to process important social information may account for differences in rhesus monkeys’ motivation and competence to establish and maintain social relationships later in life. Further development of this model will facilitate identification of novel biological targets for intervention to improve social outcomes in at-risk young monkeys.

## Introduction

Autism spectrum disorder (ASD) is a neurodevelopmental disorder characterized by core deficits in social perception and social-emotional reciprocity [1]. Despite being one of the most devastating disorders of childhood in terms of prevalence (1:68 US children) [2] and societal cost ($236B expended annually in the US) [3], there are currently no medications to treat the core social deficits of ASD. Progress has been impeded by the inability to study disease biology directly in patients and matched controls, and the fact that mice as a species lack the complex social cognition abilities most relevant to modeling ASD. These constraints underscore the tremendous value in developing novel animal models of social deficits with more reliable behavioral correlates to the human disease.

Rhesus monkeys are an ideal model organism by which to advance this objective. Like humans, they are a highly social species capable of complex social cognition. Humans and rhesus monkeys, particularly adult individuals, likewise both display stable and pronounced individual differences in social functioning. Moreover, Low-Social (LS) monkeys initiate fewer approaches and groom-presents and spend less time in conspecific proximity than do High-Social (HS) monkeys [4,5], suggesting that LS and HS monkeys differ in their motivation to interact with others. LS monkeys also receive fewer approaches, lipsmacks, and groom-presents compared to HS monkeys, indicating that LS animals may be perceived as less socially attractive [4]. Adult LS monkeys may also have poorer social perception skills. In rhesus monkeys, prolonged gaze and behaviors such as tooth-grinds and lunges can serve as aggressive signals to reinforce submissive gaze aversion [6–8]. When presented with videotapes of an unfamiliar monkey displaying such aggressive behaviors, adult HS monkeys employ a species-normative response, by quickly averting their gaze. Adult LS monkeys, in contrast, take nearly twice as long to avert their gaze compared to adult HS monkeys [9].

Social perception and social cognition deficits in children at risk for developing ASD begin to emerge by the first two years of life [10–12]. In rhesus monkeys, it is unknown how early in development the social impairments, later evident in adult LS monkeys, might emerge. It seems likely, however, that LS monkeys may begin to show impairments in social information processing early in life, as an infant’s capacity to engage in social interactions is fundamental to normative social development. In fact, the most important skills that must be acquired by an infant macaque living in a social group are the ability to recognize faces and categorize troop members into socially meaningful classes [13–14], and the ability to respond appropriately to the social signals (e.g., facial and postural gestures) of conspecifics [13–17]. In human infants, the ability to discriminate among facial identities and facial expressions emerges in a rudimentary way at 4–6 months, thereafter undergoing significant refinement during the first two years of life. These perceptual abilities continue to mature into adolescence [18]. These critical social perception and social cognition abilities ‘come on line’ in rhesus monkey infants during the first two months of life [13, 15–16, 19–21]. Infant monkeys spend their first month of life in physical contact with, or within arm’s reach of, their mother. As early as their second month of life, infant monkeys start to explore their immediate environment and to spend increasing amounts of time engaged in social interactions with other individuals within the social group. By 6 months of age, when weaning begins, the amount of time infants spend with their mothers substantially declines, whereas interactions with peers continue to increase in both frequency and complexity across development [22–23]. It is therefore reasonable to hypothesize that monkeys later classified as LS and HS may exhibit pronounced differences in social functioning by 3–4 months of age.

The goals of the present study were two-fold. First, we tested retrospectively whether monkeys classified later in life (i.e. 1–4 years of age) as LS or HS differed as infants in their ability to process important social information (e.g., face recognition memory performance and the ability to respond appropriately to conspecific social signals, including gaze aversion to aggression), using data obtained from two tests that had been administered previously as part of a colony-wide infant assessment program. Second, we tested whether summary measures of infant social abilities could predict social classification (i.e., LS vs. HS) later in life. Successful achievement of these goals could result in a high-throughput screening tool to identify monkeys that could be used to better understand the developmental and neurobiological underpinnings of social deficits implicated in ASD.

## Materials and Methods

### Subjects

Subjects were N = 164 male rhesus monkeys (*Macaca mulatta*) that were born at the California National Primate Research Center (CNPRC). Our focus on males derived from the fact that ASD is extremely male-biased in prevalence (4:1, male: female). The sample size reflects all available male subjects that were born into, and were living in, the field corrals [outdoor, half-acre (0.19 ha) cages measuring 30.5 m wide × 61 m deep × 9 m high] that were 1–4 years of age at the time of observation, and that had participated in CNPRC’s BioBehavioral Assessment program (see below). Mean (SD) age of animals was 2.28 (0.91) years with a range of 1.07 to 4.32 years. Juvenile male subjects are an ideal model to explore how low social functioning naturally develops in a population of rhesus macaques, as between 1 year of age and the onset of puberty (i.e. 5 years), they engage in social interactions at high and stable rates, and develop behavioral patterns that will be crucial for adult social functioning, including courtship and reproductive behaviors as well as dominance-aggressive interactions [24].

Each corral contained up to 180 animals of all ages and both sexes. Subjects were tattooed soon after birth and dye-marked prior to behavioral observation to facilitate easy identification. Monkeys had *ad libitum* access to Lixit-dispensed water, primate laboratory chow was provided twice daily, and fruit and vegetable supplements were provided twice weekly. Various toys, swinging perches, and other enrichments in each cage, along with outdoor housing, provided a stimulating environment. All procedures were approved by the University of California, Davis’s Institutional Animal Care and Use Committee (protocol numbers 16859 and 17192) and complied with NIH policies on the care and use of animals.

### Social Classification Determination

Subjects were observed unobtrusively in their home field corrals. Each observer conducted scan samples for a given corral during two observation periods per day (0900–1200 and 1300–1600 hr). In each observation period, scan sampling was conducted at 20 minute intervals, at a rate of 18 scans per day, for a total of five days per corral. Thus, approximately N = 90 scans were performed per corral. During each scan, the subjects in each corral were identified, and observers then recorded the occurrence of the following behaviors: non-social (subject is not within an arm’s reach of any other animal and is not engaged in play), proximity (subject is within arm’s reach of another animal), contact (subject is touching another animal in a non-aggressive manner), contact aggression (contact with another animal that is aggressive in nature, including biting, grappling, or slapping), groom (subject is engaged in a dyadic interaction with one animal inspecting the fur of the other animal using its hands or mouth), and play [subject is involved in chasing, wrestling, slapping, shoving, grabbing, or biting accompanied by a play face (wide eyes, open mouth without bared teeth) or a loose, exaggerated posture and gait; the behavior must be deemed non-aggressive to be scored]. The identities or age/sex classes (if identity could not be determined) of all social partners were recorded. Prior to behavioral data collection, the three study observers established reliabilities >85% agreement on behavioral categories, subject identities, and age/sex classes. Following completion of data collection, monkeys were rank ordered on their total frequency of non-social behavior (summarized across the 90 scan samples). The N = 25 monkeys with the greatest frequency of non-social behavior were classified as LS, and the N = 25 monkeys with the lowest frequency of non-social behavior (and therefore the highest frequency of pro-social behavior) were classified as HS. These N = 50 monkeys were subsequently enrolled in our retrospective study of infant social abilities as described below.

### Infant BioBehavioral Assessment (BBA) Program

Our N = 50 monkey subjects were previously enrolled as infants in the colony-wide BBA Program at a mean (SD) age of 106.0 (10.3) days of age (range: 90–127 days). The BBA Program consists of a battery of tests designed to assess infants' behavioral and physiological reactivity as described in detail elsewhere [25–26]. Briefly, infants were removed from their home cages and separated from their mothers for a 25 h period between 3–4 months of age. BBA testing occurred in cohorts of five to eight monkeys at a time, drawn from multiple field corrals. During testing, subjects were housed individually in standard-sized adult female holding cages (39 x 52 x 47 cm), and each infant was individually assessed according to a predetermined random order. Immediately following the completion of BBA testing, infants were reunited with their mothers, and one hour later, returned to their home corrals.

Two tests in this battery involved quantifying aspects of infant social perception and cognition, providing a means by which to test whether juvenile LS and HS monkeys differed as infants in face recognition memory performance and the ability to respond appropriately to conspecific social signals. Both tests had been video-recorded during the monkey’s infancy and were then coded at a later date, blinded to group classification. For the purpose of this study, subjects’ videotaped behavior during each test was coded using Observer XT software (Noldus Inc., Leesburg, VA, USA), while videotapes were played at 1/5^th^ speed. Coder reliabilities for each test were established prior to video coding at greater than 85% (see details below).

### Face Recognition Memory Test

#### Stimuli

This test consisted of still color photos of rhesus monkey faces projected onto a monitor (32" Panasonic KV 32540) in front of the infant subject. Stimuli were neutral faces of unfamiliar individuals of different ages (i.e., adult and juvenile) and sex (see S1 Fig). The software package ‘Cortex’ was used to program the stimuli. Once programmed, the stimulus sequence was played back on a computer monitor and recorded to create a stimulus DVD. During testing, two pictures (each measuring 19.7 x 22.9 cm) were always presented simultaneously, with each picture occupying either the left or right third of the screen. A low-light camera (Radio Shack Observation 49–2502), attached to the playback monitor and situated midway between the two projected images, was used to record the subjects’ looking responses. Each subject was administered seven problem sets, with each problem comprising one “familiarization” and two “recognition” trials. During a familiarization trial, the subject was presented with a pair of identical rhesus monkey faces. Following a delay, the participant was then presented, during the recognition trial, with the now familiar face and a novel face. Face recognition memory was inferred if subjects looked longer at the novel face compared to the familiar face. Lack of face recognition was inferred when participants looked at both face stimuli equally.

#### Procedure

**Fig 1:**
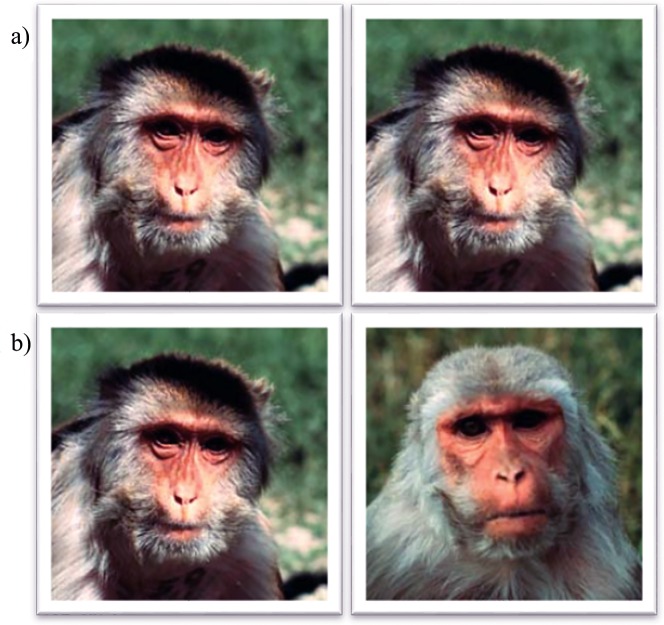
Face recognition memory test. During a familiarization trial (a) infants were presented with two identical unfamiliar rhesus monkey faces. During the subsequent recognition trial (b) infants were presented with the same rhesus monkey face from the immediately preceding familiarization trial as well as a novel face.

Infant subjects were transported from their holding cage to the test cage and given a 30-sec habituation period before the stimulus DVD began. Each subject was administered seven problem sets. Each problem set began with a 5-sec presentation of the blank (white) screen, followed by a 20-sec familiarization trial. The familiarization trial was followed by two 8-sec recognition trials, each of them separated by a 5-sec delay period. During the first recognition trial, the familiar face was presented on one side of the screen, and the novel face presented on the other side of the screen (with side presentation determined randomly) (Fig 1A and 1B). During the second recognition trial, the stimulus positions were reversed. A tone of 1000 Hz, which emanated from the monitor’s speaker, was presented 250 milliseconds prior to each trial to facilitate the subjects’ orienting to the monitor. Upon completion of testing, the subject was returned to its holding cage, and the test area cleaned and prepared for the next subject. Four measures of looking behavior were scored using Observer software from the video taken of each animal’s testing session. Intra-observer reliability was established by coding each of 26 videos on two occasions 2.5–5 months apart, and calculating the percent agreement (line by line) of the two sets of codings; reliability was 87.2%. The four measures coded for each trial were duration of gaze: 1) directed to the left stimulus, 2) directed to the right stimulus, 3) directed elsewhere (but determinable), and 4) not determinable. The duration of time gazing at the target stimulus was calculated as the sum of 1 and 2. For the recognition trials, the durations for 1 and 2, above, were recoded as familiar and novel faces, and the principal outcome measure was calculated as the proportion of time on target in which gaze was directed to the novel face summed across both recognition trials. We predicted that the two groups would not differ on duration of time spent looking at faces during the familiarization period. However, we predicted that HS, but not LS, monkeys would exhibit a greater preference for novel compared to familiar faces during recognition trials, consistent with the notion that LS monkeys have impaired face recognition memory abilities.

### Response to Conspecific Social Signals Test

#### Stimuli

A 10-min color videotape depicting an unfamiliar adult male rhesus monkey was presented to the infant subjects. The stimulus animal alternated between neutral and aggressive behavior displays (hereafter called exemplars). The videotape was created from footage obtained by placing the stimulus monkey into an aluminum cage (0.8 × 0.8 × 1.0 m) that had a clear Plexiglas front. A color camera (Panasonic AGDVC7) was placed 1.1 m in front of the cage, and camera-directed behaviors were elicited from the animals by human technicians located behind the camera. The videotape was edited into a 10-min tape, and contained 9 edits. Edits were performed to render the transition between exemplar segments as smooth as possible. Sound was present on the stimulus videotape. The neutral exemplar segments depicted the stimulus monkey displaying tactile and oral exploration of the cage, and visual exploration of the cage and surrounding area. The aggression exemplar segments depicted threats, tooth-grinds, yawns, lunges, and cage shakes. A total of four neutral (283 sec) and three aggression (317 sec) exemplar segments comprised the stimulus tape.

#### Procedure

Infant subjects were transported from their holding cage to the aluminum/Plexiglas test cage described above. After a 2-min habituation period, the stimulus videotape was played using a DVD player (Panasonic DVDS27S) and a viewing monitor (32" Panasonic KV 32540) placed 1.2 m in front of the viewing cage. The display of social stimuli using videotape permits a standardized stimulus to be presented to all animals under controlled conditions, and evidence suggests that animals respond appropriately to such presentations [27–28]. In each of the 7 exemplar segments (i.e., neutral or aggression), the following behaviors were scored: 1) gaze aversion count, measured as the number of glances made by subjects away from the video; 2) looking count, measured as the number of times the subject attended to the video; and 3) looking duration, measured as the time the subject attended to the video. All three measures were divided by the duration of the individual video segment to convert counts to rates, and the duration to the proportion of the segment, so that exemplar segments of different lengths could be properly compared. The principal outcome measure for this test was the rate of gaze aversions per minute for each exemplar segment of the video. Given that the socially appropriate response to aggression is gaze aversion, we predicted that LS and HS monkeys should differ in gaze aversion during aggression exemplars (i.e., LS monkeys will fail to exhibit species-normative gaze aversion compared to HS monkeys), but not during neutral exemplars. Looking rate is a measure of attention modulation and vigilance to aggressive stimuli, and thus we predicted that HS animals should look more frequently at the aggression exemplars, but that the HS and LS monkeys should not differ in attention to the neutral exemplar. Finally, we predicted that both groups would allocate more time to looking at the aggressive vs. the neutral exemplars, consistent with the notion that emotionally salient stimuli, especially those with a threat-related value, are particularly effective in automatically capturing the visual-attention system.

Two independent coders scored infants’ looking behaviors during the test. Inter-observer reliability was calculated for 10% of infants (N = 5). Cronbach’s alpha coefficient was used to calculate the average observer agreement for all the behaviors analyzed (looking duration: α = 0.98; looking count: α = 0.88; gaze aversion count: α = 0.88). An alpha value of 0.70 or higher is considered an acceptable reliability coefficient [29–31].

### Statistical Analysis

Data were analyzed using JMP Pro 12 for Windows (SAS Institute Inc., Cary, NC). For Mixed Models, the assumptions of Linear Mixed Models (normality of error, homogeneity of variance, and linearity) were examined graphically and suitable transformations applied as required [32]. Effect sizes for continuous outcome variables are given as partial etas ($\eta_{p}^{2}$). Where possible we used repeated measures analyses, as including subject in the analysis controls for possible extraneous sources of variance associated with subject (e.g., field corral, age, rank) within the analysis.

#### Face Recognition Memory Test

We summed the time spent on target attending to faces during the familiarization trials, and the time on target across both recognition trials, and calculated a proportion accordingly. All sessions in which a subject spent time on target in at least one of the recognition trials were included (if the animal never attended to the stimuli, a preference cannot be calculated), regardless of the time on target during familiarization. (We confirmed that this did not introduce an artefact by also analyzing these data with a controlling variable that identified trials in which the animal had not attended to the familiarization trial. This analysis yielded consistent results, thus the simpler analysis is presented here). Finally, different face stimuli problem sets may have varying motivational properties or salient features to subjects. The final data thus were calculated as the percentage of total time spent attending to faces during the familiarization trials per problem set, and as the percentage of total time on target spent attending to the novel face during the recognition trials per problem set.

These data were analyzed as a repeated measures REML mixed model, in which subject nested within social group (i.e., LS or HS) was treated as a random-effect, social group was treated as a fixed effect between-subjects experimental factor, and problem set was treated as a within-subjects fixed effect blocking factor. The interaction of trials-by-social group was tested to detect and control for any problem sets that might systematically confound the result. A significant difference due to social group would only be meaningful if one or both groups also significantly differed from a 50/50 preference. This was tested *post hoc* by constructing Bonferroni-corrected (i.e. 97.5%) confidence intervals for each group. Suitable error terms for mixed model repeated measures analyses were specified as needed [33].

#### Response to Conspecific Social Signals Test

In this task, each subject watched alternating neutral and aggressive behavior exemplars. These data were analyzed as a repeated measures REML mixed model, in which subject nested within social group was treated as a random-effect, and social group was treated as a fixed effect between-subjects experimental factor. To explicitly represent the fact that there were two kinds of video exemplars, exemplar number (1 to 7) was nested within exemplar type (neutral or aggression). Exemplar type thus tests for overall effects of the type of behavior performed, and as before, the exemplar number tests whether there is variation in subjects’ response to particular exemplars (including whether the first exemplar should be treated differently being a baseline trial). These factors were treated as within-subjects fixed effects. To test the prediction that subjects of different social groups would respond differently to the different exemplar types, we tested the social group—by—exemplar type interaction. Post hoc tests were performed as Bonferroni-corrected planned orthogonal contrasts of this interaction. Suitable error terms for mixed model repeated measures analyses were specified as needed [32]. The same analysis was used for the rate of gaze aversion, the rate of looking, and the percentage of time spent looking at each exemplar type. The rate of gaze aversion was square-root transformed to meet statistical assumptions.

#### Predicting Later Social Classification

To test whether infant social information processing abilities predict later social classification, we used the output from the previous analyses to yield fitted means for each subject for each of the five measures (i.e., mean preference for a novel face, mean rate of gaze aversions to aggressive and neutral exemplars, and mean rate of looking at aggressive and neutral exemplars). Initial analyses revealed that in the response to conspecific social stimuli test, the neutral exemplars were predictive only because they (as intended) allowed each animal to be its own control for the aggressive exemplars. Therefore we simplified the data further, by calculating the ratio of looking to aggressive exemplars/looking to neutral exemplars, and calculated the same ratio for gaze aversion. We then included the resulting three measures (preference for novel faces and the two aggressive/neutral ratios) in a logistic regression model. Although this model perfectly predicted later social classification of all 50 monkeys, only the most predictive measure was actually statistically significant. Therefore, while we present the results from this logistic regression, we ultimately performed and present results from simple logistic regressions on each predictor separately to convey the significance and relative strength of each measure.

## Results

Raw data and analyses are provided as SAS code in S1 Dataset.

### Face Recognition Memory Test

**Fig 2:**
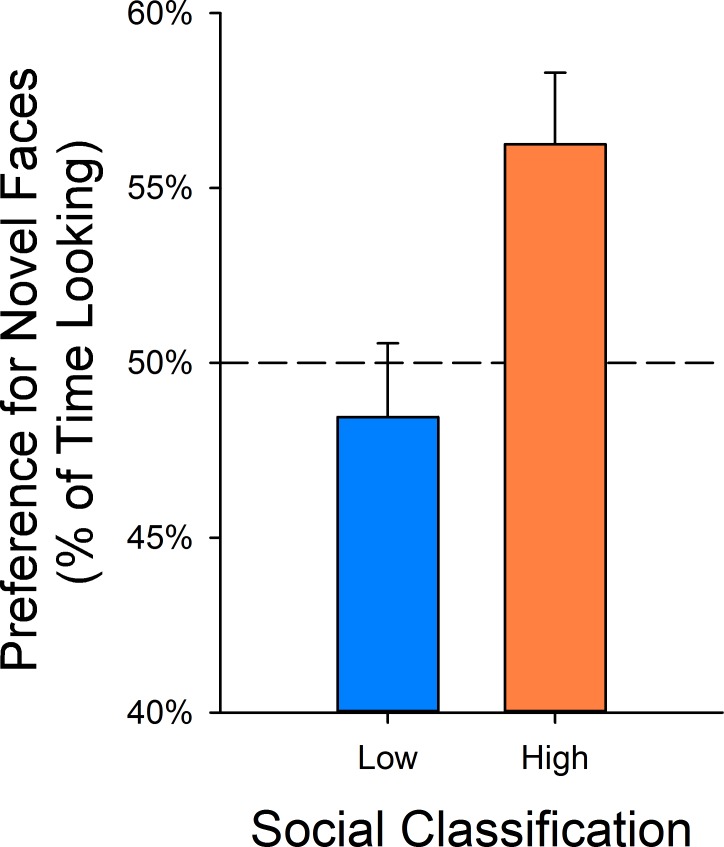
Preference for novel faces on the face recognition memory test. During recognition trials, infants later classified as Low-Social (LS) did not show a preference above chance for the novel face (percentage of time looking on target directed to the novel face) (97.5% CI: 43.6% - 53.3%); whereas infants later classified as High-Social (HS) did (97.5% CI: 51.6% - 61.0%). Data are plotted as LSM +/- SE. Effect size is given in the text as partial eta ($\eta_{p}^{2}$).

LS and HS monkeys did not differ in time spent looking at faces during the familiarization trials (F_1,48_ = 3.65; $\eta_{p}^{2}$ = 0.071; P = 0.0619). However, HS monkeys showed a greater preference for novel faces compared to LS monkeys in infancy (F_1,46.19_ = 7.04; $\eta_{p}^{2}$ = 0.132; P = 0.0109) (Fig 2). Post-hoc tests showed that LS monkeys did not differ from a 50–50 preference for novel faces (97.5% CI: 43.6% - 53.3%), while HS monkeys exhibited a significant preference for novel faces (97.5% CI: 51.6% - 61.0%). The mean preference for novel faces did differ between problem sets (F_6,271.9_ = 4.25; $\eta_{p}^{2}$ = 0.086; P = 0.0004), indicating the importance of controlling for this variable. There was no interaction between problem set and social group (F_6,271.9_ = 0.40; $\eta_{p}^{2}$ = 0.009; P = 0.8758), indicating that no particular problem set was driving or confounding the overall effect of social group.

### Response to Conspecific Social Stimuli Test

**Fig 3:**
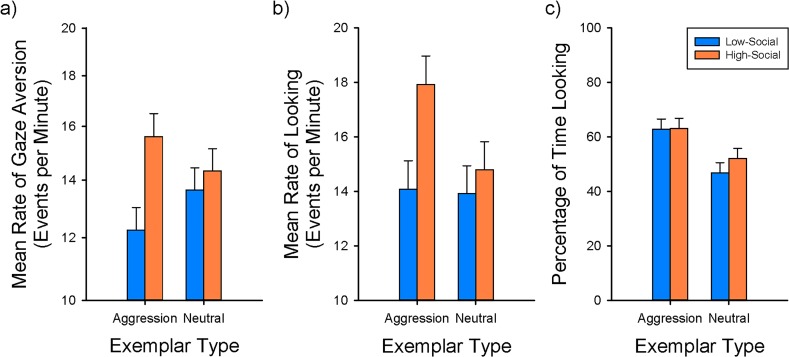
Visual attention distribution during the response to conspecific social stimuli test. Infants later classified as Low-Social (LS) showed a lower rate of gaze aversion to aggression, and looked at the social stimuli less frequently than infants later classified as High-Social (HS), but both monkey groups spent a greater percentage of time looking at aggressive vs. neutral behavioral displays. (a) The rate of gaze aversion differed between infants later classified as LS and HS only during the aggression exemplars. (b) The rate of looking also differed between infants later classified as LS and HS only during the aggression exemplars. Infants later classified as HS, but not LS, differed in their rate of looking between aggression and neutral exemplar types. (c) The percentage of time spent looking at aggression did not differ between monkey groups, and both groups spent more time looking at aggressive displays over neutral ones.

As predicted, HS and LS monkeys differed in their gaze aversion response to the exemplar types (social group—by—exemplar type interaction: F_1,48.19_ = 9.16; $\eta_{p}^{2}$ = 0.160; P = 0.0040) (Fig 3A). Orthogonal planned contrasts showed that this was due to a difference in the response of HS and LS monkeys to the aggression exemplars (F_1,68.81_ = 8.43; $\eta_{p}^{2}$ = 0.109; P = 0.0050; Bonferroni-corrected critical alpha = 0.0125), whereby HS monkeys gaze averted more; all other planned contrasts were non-significant. As one might expect, subjects reacted to the individual exemplars differently (F_5,239.8_ = 12.42; $\eta_{p}^{2}$ = 0.206; P < 0.0001), but these differences did not vary between social groups (F_5,239.8_ = 1.13; $\eta_{p}^{2}$ = 0.023; P = 0.3463), indicating that no individual video segment confounded or drove the main result.

Also as predicted, HS and LS monkeys differed in their rate of looking to the exemplar types (social group—by—exemplar type interaction: F_1,48.2_ = 15.46; $\eta_{p}^{2}$ = 0.243; P = 0.0003) (Fig 3B). Post hoc orthogonal planned contrasts showed that this was due to a difference in the response of HS and LS monkeys to the aggression exemplars (F_1,57.46_ = 6.68; $\eta_{p}^{2}$ = 0.104; P = 0.0123; Bonferroni-corrected critical alpha = 0.0125) whereby HS monkeys looked more, but no group difference was observed for neutral exemplars. In addition HS monkeys directed higher rates of looking at aggression exemplars compared to neutral exemplars (F_1,48.2_ = 34.41; $\eta_{p}^{2}$ = 0.417; P < 0.0001; Bonferroni-corrected critical alpha = 0.0125), but LS monkeys did not differ in their rates of looking at exemplar types. The individual exemplars did show overall differences (F_5,240.5_ = 14.12; $\eta_{p}^{2}$ = 0.227; P < 0.0001), as in the prior result for gaze aversion, but these differences did not vary between the social groups (F_5,240.5_ = 1.31; $\eta_{p}^{2}$ = 0.0.027; P = 0.2612), indicating that no individual exemplar confounded or drove the main result.

By contrast, for the total percentage of time spent looking at each exemplar type, HS and LS monkeys did not differ (social group—by—exemplar type interaction: F_1,48.09_ = 3.56; $\eta_{p}^{2}$ = 0.069; P = 0.0651) (Fig 3C); both HS and LS monkeys consistently spent more time looking at aggressive exemplars than at neutral exemplars (F_1,48.09_ = 105.00; $\eta_{p}^{2}$ = 0.686; P < 0.0001). The individual exemplars again showed overall differences (F_5,238.6_ = 8.91; $\eta_{p}^{2}$ = 0.157; P < 0.0001), but these differences did not vary between the social groups (F_5,238.6_ = 0.47; $\eta_{p}^{2}$ = 0.010; P = 0.7911), indicating that no individual exemplar confounded or drove the main result.

### Predicting Later Social Classification

A multivariate logistic regression analysis perfectly predicted later social classification for all monkeys (model likelihood ratio (LR) Chi-squared = 69.28; DF = 3; P < 0.0001). However, given the resulting quasi-complete separation of the model, and the correlation of the three predictors, only the most powerful predictor was significant (rate of looking aggression / neutral ratio; LR Chi-squared = 7.85; P = 0.0050). This did not represent the full story in the data, and thus, univariate logistic regressions were run for each predictor.

**Fig 4:**
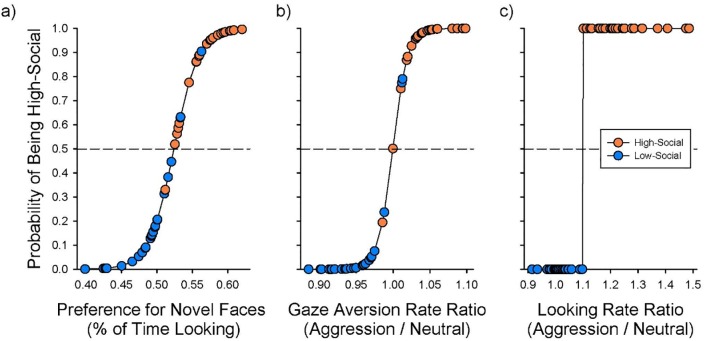
Infant social information processing abilities significantly predict social classification later in life. Given the high inter-correlation of these scores, each predictor is presented as a separate univariate logistic regression. Each subject is plotted as the predicted probability of being HS, and the data point colored for the actual classification. (a) % preference for a novel face; (b) ratio of gaze averts aggression / neutral; (c) ratio of looks aggression / neutral.

In a univariate model, mean % preference for a novel face correctly predicted 44/50 infant monkeys’ later social classification (LR Chi-squared = 36.22; P < 0.0001) (Fig 4A). Similarly, the ratio of gaze aversion to aggression / neutral exemplars correctly predicted 47/50 infant monkeys’ later social classification (LR Chi-squared = 55.63; P < 0.0001) in a univariate model (Fig 4B). Finally, the ratio of looks to aggression / neutral exemplars perfectly predicted 50/50 infant monkeys’ later social classification (LR Chi-squared = 69.31; P < 0.0001) in a univariate model (Fig 4C).

## Discussion

The goals of this study were to test retrospectively whether juvenile LS and HS monkeys differed as infants in their early ability to process social information, and whether infant social abilities could predict later social classification (i.e., LS vs. HS). LS monkeys in infancy did not differ from chance in their performance on a face recognition memory test and likewise did not exhibit species-normative responses to conspecific social signals. Individual differences in infant social information processing likewise were powerful predictors of subsequent social classification (LS or HS). These findings suggest that an early capacity to appropriately process social information and respond accordingly may account for differences in monkeys’ motivation and competence to establish and maintain social relationships later in life.

Face recognition memory in this study was assessed using a visual paired-comparison (VPC) habituation paradigm which relied on differential visual attention paid to unfamiliar (i.e., novel) and familiar face stimuli following a period of familiarization [34–35]. Unequal distribution of social attention to a novel over a previously exposed target therefore indicates both discriminative ability and recognition memory on the part of the infant already in the first months of life [36–42]. It could be argued that the recognition process measured by the Face Recognition Memory Test is comparable to that which individuals use in everyday life when exploring stimuli in their environment. To master complex and changing social interactions, infants need to effectively process salient information by selectively attending to novel stimuli. Our findings indicate that infants later classified as LS lacked this capacity to discriminate a novel from a familiar stimulus, thereby suggesting an early impairment in recognizing conspecific faces. This finding is particularly interesting in light of well documented evidence (detailed below) that 2-year-old children at familial risk for developing ASD, and school-aged children diagnosed with it, show robust deficits in the ability to recognize human faces [43–46].

Unlike typically developing subjects, children with ASD appear to have difficulties in encoding facial identity [43–45], and perform worse on face recognition tasks, compared to chronological and mental verbal age-matched children with learning disabilities [46]. These deficits in face recognition persist across lifespan development, as adolescents and adults with ASD likewise show impaired face recognition [47–51]. Several, but not necessarily mutually exclusive, hypotheses could be advanced to explain the impaired face recognition skills found in our study. Similar to what has been suggested for children with ASD [52–53], it could be argued that the impairment in face recognition found in LS monkeys originates from a failure to attend to face stimuli. However, in contrast with this account, our two groups of infant monkeys did not differ in the amount of time spent looking at the stimuli during the familiarization period. Nonetheless, only infants later classified as HS showed a preference for novel faces during the recognition trials. Thus, the enhanced face recognition abilities shown by HS subjects cannot be explained by a difference in visual attention to the familiar stimuli.

Differences in LS and HS infant face recognition abilities could also be linked with the employment of different face processing strategies. In fact, an important aspect of face processing observed in both monkeys and humans pertains to facial feature analysis. The analysis of scan-paths in human and some non-human primate species indicates that, when looking at faces with neutral expressions, all primates tend to spend more time looking at the eyes than at any other facial feature (e.g. mouth, ears, forehead, neck), and rely heavily on the eyes to discriminate between the faces of individuals [54–63]. Robust visual attention directed towards the eye region also appears to be indicative of holistic face processing [62–63]. Human infants as early as 4–7 months of age likewise vary in how they distribute attention to face stimuli [64–65], and variation in attentional bias is associated with variation in face recognition. A large body of evidence indicates that infants who tend to process global properties before local properties, much as adults do, process information faster and more efficiently than infants who tend to focus initially on local aspects of the stimuli [66–72]. Moreover, several studies have shown abnormalities in the visual scanning of faces by individuals with ASD [73–76]. When scanning dynamic social scenes, both 15-month-old infant and older children with ASD showed heightened scanning of the mouth and body parts, at the expense of scanning of the eyes [73, 77]. Furthermore, Pelphrey and colleagues [73] found that, when scanning static images of faces, adults with ASD tended to spent more time scanning the non-feature areas (e.g., ears, chin) and spent less time scanning feature areas (i.e., eyes) than controls.

Considering that the distribution of attention within a visual scene reflects an active process of gathering relevant information [78–80], one explanation for the group difference found in visual attention directed towards novel faces in the present study could be that LS monkeys used a different scanning strategy compared to HS monkeys. Although this explanation is only speculative, and would require the use of eye-tracking technology in order to objectively measure infants’ attention distribution, it is possible that LS monkeys may have visually attended to less salient features of the face, and this could account for their impairments in processing faces efficiently. This early social attention deficit in attending to and processing social information may have wide-ranging ramifications, serving to deprive the developing rhesus infant of the social information that is critical for the maturation of neural connections responsible for species-typical social interactions [81]. In this sense, visual attention distribution and recognition memory might constitute two aspects of early information processing that have important implications for social development.

Another important aspect of infant social information processing is the ability to perceive and respond to different facial expressions. In fact, the second aim of this study tested infants’ gaze distribution as they watched a videotape containing both neutral and aggressive social signals. Previous research has suggested that human infants are sensitive to different facial expressions already in the first months of life [82–83], and showed also distinct brain responses to emotional facial expressions beginning at 4 months of age [84–87]. Several studies have shown that threatening faces are detected more quickly and accurately than friendly faces, strengthening the idea that social threat captures attention [88–90]. In line with this, the current study revealed that macaque infants’ looking behavior varied in response to different socio-emotional displays by a stimulus monkey. In fact, both groups of infants showed an increased duration of visual attention during aggressive compared to neutral exemplars. It seems reasonable to assume that the appearance of potentially dangerous stimuli may be particularly effective in capturing the visual-attention system, as it is highly adapted to rapidly detect and respond to threat-related stimuli. Evidence comes from several studies showing that, in both human and non-human primates, fear relevant stimuli such as snakes, spiders, and angry faces might have a biological basis for being prioritized by the attentional system [91–96], and may function as “innate releasing stimuli” even very early in life [19]. Our findings are consistent with this notion.

Moreover, threat-related and neutral/positive emotional expressions elicit distinct visual scanning behaviors in both infant and adult individuals [97]. Indications for distinct scanning patterns for facial expressions of threat-related and neutral emotions have been previously described in both non-human [60, 98–100] and human [97, 100] primates. Several studies have reported that angry faces are detected rapidly [101–102], but are subsequently avoided [101, 103]. Gaze aversion when confronted with an angry individual is a submissive act, which avoids conflict, and orienting away from the eyes might, in turn, relieve the individual’s emotional distress [99, 104]. Such a strategy may have an adaptive value, as it prevents or reduces aggression, and increases social approach [105–107]. In agreement with these findings, our results indicated that threat-related stimuli did elicit different visual attention patterns in the two groups of infants. We found that infants later classified as HS showed species-typical attentional disengagement in response to aggressive scenes compared to infants that were later classified as LS. During testing, HS infants’ rate of gaze aversion increased as well as their rate of looking, reflecting heightened vigilance for threatening social situations, as described also in earlier studies with 3–6 year-old children and adult humans [100, 108]. LS infants, in contrast to HS infants, did not differentially modulate visual attention across the aggressive and neutral video segments. Instead, their gaze distribution pattern during the aggression segments was characterized by a lower rate of gaze aversion as well as a lower rate of looking compared to HS infants. This latter finding suggests that LS infants might have difficulties in responding appropriately to rapidly changing social contexts. Since adult LS monkeys also exhibit impairments in species normative gaze aversion to conspecific aggression [4], the origin of poor social skills demonstrated by adult LS monkeys may likely have its origins in early infancy. In human children, deficits in social interaction and the ability to understand and appropriately respond to others’ actions and emotional states is a diagnostic feature of ASD [1]. In fact, in addition to abnormalities in visual scanning of faces, children with ASD also perform worse when trying to recognize other people’s emotional expressions [109–110]. When looking at faces showing emotional expressions, children with ASD look less at the feature areas (eyes, mouth) compared to typically developed children [10, 108]. Interestingly, there is some evidence showing that people with ASD may have particular deficits in processing negative basic emotions, such as fear [74, 111], anger [112–113], and disgust [114]. However, there is mixed evidence regarding whether individuals with ASD are impaired in the recognition of basic emotions, with multiple studies reporting that individuals with ASD do not show atypical recognition of basic emotional expressions, especially in the case of high-functioning individuals [115–118]. Some studies, using a facial visual search paradigm, have shown that people with ASD are better able to detect threatening than friendly faces, although more slowly and with less accuracy than control individuals [119–120]. Nonetheless, the ability to implicitly process threat does not provide evidence for the ability to explicitly respond to threat. Similarly, our results showed that both groups of infants were able to detect the presence of a threatening stimulus, but the ability to modulate the gaze and respond appropriately to different stimuli was observed to a much lesser degree in LS animals. It is possible that basic innate mechanisms allow rapid detection of threatening facial expressions. However, although able to implicitly process basic emotions, LS animals may not be able to determine the mental state associated with certain emotional facial expressions. This inability may lead to impairments in social interaction rather than an inability to detect social threat in general.

Finally, although these findings are congruent with the hypothesis that LS infant monkeys have difficulty responding properly to social stimuli, these data also raise the possibility that LS infant monkeys may have more general problems with responsiveness (orientation/attention) to all types of sensory stimuli. Interestingly, recent studies have shown that differences in visual preference toward social *vs*. non-social stimuli are already evident in newborns at low and high risk for ASD, with the latter group being more attentive to non-social stimuli [121]. Our available data, unfortunately, do not help us differentiate between these two possibilities. Further study is needed to determine whether the impairments seen in LS monkeys are specifically related to their social competence, or rather, their more general cognitive disabilities.

Although caution should be exercised in labelling LS monkeys as “pathological”, our data strongly suggest that these animals performed poorly on both tasks, thus revealing significant impairments in their social functioning. Indeed, LS animals did not differ from chance in their ability to recognize familiar faces on the Face Recognition Memory Test. Previous studies of infant monkeys with damage to limbic structures [122], exposed prenatally to methyl mercury [123], and from high-risk pregnancies and births [124] have shown that these individuals direct a significantly lower proportion of their looking time to novel stimuli than control subjects. Similarly, human infants at risk for intellectual impairment do not show a visual preference for novel faces, whereas typically developing infants do (for a review see [125]). Considered collectively, this body of evidence indicates that, although our subjects were selected from the social extremes of a distribution, HS animals nevertheless performed normatively on the Face Recognition Memory Test, whereas LS animals did not.

Similarly, in response to conspecific social stimuli, LS subjects failed to exhibit the adaptive species-typical coping response to aggression. Our results from infant rhesus monkeys complement previous research findings from adult rhesus monkeys, indicating that these early social impairments persist into adulthood. Indeed, adult LS animals also exhibit longer latencies to gaze avert to aggressive displays [4]. It is possible that, by virtue of their inability to cope properly with different social situations, LS individuals may experience the world as unusually threatening, which in turn, may contribute to high levels of social avoidance. This hypothesis would also be in agreement with data indicating that LS adult monkeys have a lower reward sensitivity [126–127] and interest in novelty [128] compared to HS animals, as well as a more maladaptive pattern of physiological activation [4, 9, 127, 129–133]. Nonetheless, further investigation of LS monkey performance on social functioning measures with direct relevance to ASD is needed in order to define LS animals as a pathological, socially impaired group.

In conclusion, the current study provides the first evidence that variation in the ability to recognize conspecific faces and respond appropriately to social signals differentiate infants at risk for poor social outcomes (i.e., those later classified as LS). Our findings showed that social information processing abilities in infancy might be necessary requirements for optimal social functioning and development, especially as these abilities were strongly predictive of subsequent social classification. These very early social information processing abilities likely constitute the building blocks for the acquisition of more complex social abilities at later ages. Individual differences in the early capacity to process important social information may, in fact, account for differences in monkeys’ motivation and competence to establish and maintain social interactions later in life. Our results also suggest that deficits in early information processing can be detected within the first months of life, and that the trajectories from infant social attentional abilities to later complex social cognition abilities can, in principle, be delineated. Moreover, by 3–4 months of age, the pattern of behaviors evident in monkeys later classified as LS was contrastingly different from the patterns displayed by those later classified as HS. By implication, measures of infant social information processing may hold promise as early screening assessments to identify monkeys at risk for poor social outcomes. Further research would allow for identification of biological targets and development of an early-intervention therapeutic testing pipeline to improve social attention in at-risk monkeys during infancy.

## Supporting Information

S1 Dataset Face Recognition Memory Test and Response to Conspecific Social Signals Test raw data and analyses. (DOCX)

S1 Fig Face Recognition Memory Test Stimuli. (PDF)
