# Supplementary material for: Early Predictors of Impaired Social Functioning in Male Rhesus Macaques (Macaca mulatta)
Source: PLoS One. 2016 Oct 27;11(10):e0165401. doi: 10.1371/journal.pone.0165401 (PMC5082922; doi:10.1371/journal.pone.0165401)
Supplement: S1 Dataset — (DOCX) [file pone.0165401.s001.docx]

# Supplementary data

The raw data for each of the analyses are presented below. Each data set is given as a SAS code for the data itself, and the equivalent analysis to that performed in JMP (and reported in the text). Data are presented in SAS format as this is a simple text format. The data and code were generated as direct exports from JMP, and additional SAS code added as needed (for instance, JMP does not export code for post-hoc tests).

# Face Recognition Memory Test

## Duration of Looking in Familiarization Trials

**DATA** PL_task_repeated_measures_forma; INPUT ANIMID SociabilityText &$16. Session sqrt_DurFamil_ToT; Lines;

**40737** Low-Sociable **1** **0.448330235429198**

**40737** Low-Sociable **2** **0.602346477535978**

**40737** Low-Sociable **3** **0.561877928023517**

**40737** Low-Sociable **4** **0.251992063367083**

**40737** Low-Sociable **5** **0.403112887414928**

**40737** Low-Sociable **6** **0.274584822960046**

**40737** Low-Sociable **7** **0.707106781186548**

**41470** Low-Sociable **1** **0.610737259384099**

**41470** Low-Sociable **2** **0.33166247903554**

**41470** Low-Sociable **3** **0.272029410174709**

**41470** Low-Sociable **4** **0.385356977359954**

**41470** Low-Sociable **5** **0.168819430161341**

**41470** Low-Sociable **6** **0.403732584763727**

**41470** Low-Sociable **7** **0.603738353924943**

**41475** High-Sociable **1** **0.433052860514741**

**41475** High-Sociable **2** **0.285482048472404**

**41475** High-Sociable **3** **0.226936114358204**

**41475** High-Sociable **4** **0.14142135623731**

**41475** High-Sociable **5** **0.376164857476081**

**41475** High-Sociable **6** **0.187082869338697**

**41475** High-Sociable **7** **0.128452325786651**

**41518** High-Sociable **1** **0.646915759585435**

**41518** High-Sociable **2** **0.562107216641096**

**41518** High-Sociable **3** **0.806225774829855**

**41518** High-Sociable **4** **0.308220700148449**

**41518** High-Sociable **5** **0.665206734782504**

**41518** High-Sociable **6** **0.797809501071528**

**41518** High-Sociable **7** **0.958384056628657**

**41520** Low-Sociable **1** **0.440823117587996**

**41520** Low-Sociable **2** **0.345687720348872**

**41520** Low-Sociable **3** **0.39560080889705**

**41520** Low-Sociable **4** **0.14142135623731**

**41520** Low-Sociable **5** **0.208566536146142**

**41520** Low-Sociable **6** **0.197484176581315**

**41520** Low-Sociable **7** **0.215638586528478**

**41556** High-Sociable **1** **0.792464510246358**

**41556** High-Sociable **2** **0.701070609853244**

**41556** High-Sociable **3** **0.477493455452533**

**41556** High-Sociable **4** **0.659545297913646**

**41556** High-Sociable **5** **0.504975246918104**

**41556** High-Sociable **6** **0.407069153830157**

**41556** High-Sociable **7** **0.761577310586391**

**41569** Low-Sociable **1** **0.424264068711929**

**41569** Low-Sociable **2** **0.33689761055846**

**41569** Low-Sociable **3** **0.237697286480094**

**41569** Low-Sociable **4** **0.212132034355964**

**41569** Low-Sociable **5** **0.146628782986152**

**41569** Low-Sociable **6** **0.347131099154196**

**41569** Low-Sociable **7** **0.153297097167559**

**41659** Low-Sociable **1** **0.708872343937891**

**41659** Low-Sociable **2** **0.63521649852629**

**41659** Low-Sociable **3** **0.722841614740048**

**41659** Low-Sociable **4** **0.526307894677631**

**41659** Low-Sociable **5** **0.706045324324154**

**41659** Low-Sociable **6** **0.521056618804521**

**41659** Low-Sociable **7** **0.571401785086466**

**41662** Low-Sociable **1** **0.3**

**41662** Low-Sociable **2** **0.207364413533277**

**41662** Low-Sociable **3** **0.255929677841395**

**41662** Low-Sociable **4** **0.355668384875575**

**41662** Low-Sociable **5** **0.220009670242015**

**41662** Low-Sociable **6** **0.263628526529281**

**41662** Low-Sociable **7** **0.313049516849971**

**42039** Low-Sociable **1** **0.668206554891525**

**42039** Low-Sociable **2** **0.280178514522438**

**42039** Low-Sociable **3** **0.336154726279432**

**42039** Low-Sociable **4** **0.237697286480094**

**42039** Low-Sociable **5** **0.173205080756888**

**42039** Low-Sociable **6** **0.0707106781186548**

**42039** Low-Sociable **7** **0.168819430161341**

**42127** High-Sociable **1** **0.966953980290686**

**42127** High-Sociable **2** **0.719722168617863**

**42127** High-Sociable **3** **0.856446145417212**

**42127** High-Sociable **4** **0.815781833580523**

**42127** High-Sociable **5** **0.936215787091844**

**42127** High-Sociable **6** **0.74128267212987**

**42127** High-Sociable **7** **0.61400325732035**

**42156** High-Sociable **1** **0.690289794216893**

**42156** High-Sociable **2** **0.478016736108685**

**42156** High-Sociable **3** **0.369459064038223**

**42156** High-Sociable **4** **0.467974358271904**

**42156** High-Sociable **5** **0.338378486313773**

**42156** High-Sociable **6** **0.39560080889705**

**42156** High-Sociable **7** **0.399374510954317**

**42183** Low-Sociable **1** **0.694982014155762**

**42183** Low-Sociable **2** **0.668206554891525**

**42183** Low-Sociable **3** **0.673795221116921**

**42183** Low-Sociable **4** **0.430116263352131**

**42183** Low-Sociable **5** **0.367423461417477**

**42183** Low-Sociable **6** **0.223606797749979**

**42183** Low-Sociable **7** **0.360555127546399**

**42283** Low-Sociable **1** **0.689560571668653**

**42283** Low-Sociable **2** **0.458802789878178**

**42283** Low-Sociable **3** **0.271108834234519**

**42283** Low-Sociable **4** **0.466368952654441**

**42283** Low-Sociable **5** **0.459347363114234**

**42283** Low-Sociable **6** **0.564843003851513**

**42283** Low-Sociable **7** **0.53840593607426**

**42299** Low-Sociable **1** **0.76909037180295**

**42299** Low-Sociable **2** **0.606217782649107**

**42299** Low-Sociable **3** **0**

**42299** Low-Sociable **4** **0.254950975679639**

**42299** Low-Sociable **5** **0.590338885725818**

**42299** Low-Sociable **6** **0.292403830344269**

**42299** Low-Sociable **7** **0.711688133946323**

**42324** High-Sociable **1** **0.760448221116994**

**42324** High-Sociable **2** **0.619273768215641**

**42324** High-Sociable **3** **0.909893937225653**

**42324** High-Sociable **4** **0.678337724146313**

**42324** High-Sociable **5** **1**

**42324** High-Sociable **6** **0.472166470643564**

**42324** High-Sociable **7** **0.234520787991172**

**42333** High-Sociable **1** **0.877605205089396**

**42333** High-Sociable **2** **0.527730992078351**

**42333** High-Sociable **3** **0.486826457785523**

**42333** High-Sociable **4** **0**

**42333** High-Sociable **5** **0.384707681233427**

**42333** High-Sociable **6** **0**

**42333** High-Sociable **7** **0.612372435695795**

**42369** High-Sociable **1** **0.555877684387492**

**42369** High-Sociable **2** **0.551815186452856**

**42369** High-Sociable **3** **0.47169905660283**

**42369** High-Sociable **4** **0.468508271004899**

**42369** High-Sociable **5** **0.297209241108011**

**42369** High-Sociable **6** **0.424264068711929**

**42369** High-Sociable **7** **0.569649014745045**

**42371** Low-Sociable **1** **0.583523778435806**

**42371** Low-Sociable **2** **0.435889894354067**

**42371** Low-Sociable **3** **0.503487835006964**

**42371** Low-Sociable **4** **0**

**42371** Low-Sociable **5** **0.412310562561766**

**42371** Low-Sociable **6** **0.122474487139159**

**42371** Low-Sociable **7** **0.454422710700071**

**42373** Low-Sociable **1** **0.543139024560011**

**42373** Low-Sociable **2** **0.355668384875575**

**42373** Low-Sociable **3** **0.535256947642905**

**42373** Low-Sociable **4** **0**

**42373** Low-Sociable **5** **0.122474487139159**

**42373** Low-Sociable **6** **0.230217288664427**

**42373** Low-Sociable **7** **0.350713558335004**

**42396** Low-Sociable **1** **0.49598387070549**

**42396** Low-Sociable **2** **0.418330013267038**

**42396** Low-Sociable **3** **0.378813938497516**

**42396** Low-Sociable **4** **0.494974746830583**

**42396** Low-Sociable **5** **0.232379000772445**

**42396** Low-Sociable **6** **0.248997991959775**

**42396** Low-Sociable **7** **0.702139587261678**

**42399** High-Sociable **1** **0.393590262836875**

**42399** High-Sociable **2** **0.465768065672176**

**42399** High-Sociable **3** **0.588642506110457**

**42399** High-Sociable **4** **0.423083916026124**

**42399** High-Sociable **5** **0.146628782986152**

**42399** High-Sociable **6** **0.418330013267038**

**42399** High-Sociable **7** **0.5**

**42402** Low-Sociable **1** **0.600832755431992**

**42402** Low-Sociable **2** **0.608393909404097**

**42402** Low-Sociable **3** **0.14142135623731**

**42402** Low-Sociable **4** **0.857321409974112**

**42402** Low-Sociable **5** **0.560802995712398**

**42402** Low-Sociable **6** **0**

**42402** Low-Sociable **7** **0.107238052947636**

**42612** Low-Sociable **1** **0.602909611799314**

**42612** Low-Sociable **2** **0.459891291502677**

**42612** Low-Sociable **3** **0.3286335345031**

**42612** Low-Sociable **4** **0.291547594742265**

**42612** Low-Sociable **5** **0.122474487139159**

**42612** Low-Sociable **6** **0.460434577328854**

**42612** Low-Sociable **7** **0.576194411635517**

**42672** High-Sociable **1** **0.462060602085917**

**42672** High-Sociable **2** **0.304959013639538**

**42672** High-Sociable **3** **0.568330889535313**

**42672** High-Sociable **4** **0.365376518128902**

**42672** High-Sociable **5** **0.0921954445729289**

**42672** High-Sociable **6** **0.168819430161341**

**42672** High-Sociable **7** **0.4**

**42694** Low-Sociable **1** **0.360555127546399**

**42694** Low-Sociable **2** **0.228035085019828**

**42694** Low-Sociable **3** **0.220227155455452**

**42694** Low-Sociable **4** **0.107238052947636**

**42694** Low-Sociable **5** **0.0806225774829855**

**42694** Low-Sociable **6** **0.32434905888564**

**42694** Low-Sociable **7** **0.519615242270663**

**42705** Low-Sociable **1** **0.74027022093287**

**42705** Low-Sociable **2** **0.532447180478965**

**42705** Low-Sociable **3** **0.33689761055846**

**42705** Low-Sociable **4** **0.294957624075053**

**42705** Low-Sociable **5** **0.221359436211787**

**42705** Low-Sociable **6** **0.151657508881031**

**42705** Low-Sociable **7** **0.114017542509914**

**42717** Low-Sociable **1** **0.529622507074614**

**42717** Low-Sociable **2** **0.2**

**42717** Low-Sociable **3** **0.226936114358204**

**42717** Low-Sociable **4** **0.134164078649987**

**42717** Low-Sociable **5** **0.162788205960997**

**42717** Low-Sociable **6** **0.219089023002066**

**42717** Low-Sociable **7** **0**

**42723** High-Sociable **1** **0.619795402532158**

**42723** High-Sociable **2** **0.512835256198323**

**42723** High-Sociable **3** **0.630079360080935**

**42723** High-Sociable **4** **0.72145685941711**

**42723** High-Sociable **5** **0.601664358259653**

**42723** High-Sociable **6** **0.772981241687015**

**42723** High-Sociable **7** **0.765832879941832**

**42725** High-Sociable **1** **0.508920425999979**

**42725** High-Sociable **2** **0.49295030175465**

**42725** High-Sociable **3** **0.470106370941726**

**42725** High-Sociable **4** **0.240831891575846**

**42725** High-Sociable **5** **0.191049731745428**

**42725** High-Sociable **6** **0**

**42725** High-Sociable **7** **0.251992063367083**

**42858** Low-Sociable **1** **0.476445169982864**

**42858** Low-Sociable **2** **0.469574275274956**

**42858** Low-Sociable **3** **0.348568879850167**

**42858** Low-Sociable **4** **0.373496987939662**

**42858** Low-Sociable **5** **0.271108834234519**

**42858** Low-Sociable **6** **0.441326911710582**

**42858** Low-Sociable **7** **0.528387363588495**

**42979** High-Sociable **1** **0.458115204943036**

**42979** High-Sociable **2** **0.668580586017871**

**42979** High-Sociable **3** **0.594138031100518**

**42979** High-Sociable **4** **0.695341642647699**

**42979** High-Sociable **5** **0.307635930931353**

**42979** High-Sociable **6** **0.577494588719236**

**42979** High-Sociable **7** **0.187616630392937**

**43131** Low-Sociable **1** **0.940744386111339**

**43131** Low-Sociable **2** **0.826438140455776**

**43131** Low-Sociable **3** **0.477493455452533**

**43131** Low-Sociable **4** **0.85146931829632**

**43131** Low-Sociable **5** **0.484767985741633**

**43131** Low-Sociable **6** **0.532447180478965**

**43131** Low-Sociable **7** **0.33166247903554**

**43157** Low-Sociable **1** **0.843504593941254**

**43157** Low-Sociable **2** **0.699285349481884**

**43157** Low-Sociable **3** **0.680073525436772**

**43157** Low-Sociable **4** **0.729040465269247**

**43157** Low-Sociable **5** **0.251992063367083**

**43157** Low-Sociable **6** **0.0921954445729289**

**43157** Low-Sociable **7** **0.18165902124585**

**43215** High-Sociable **1** **0.620886463051016**

**43215** High-Sociable **2** **0.339116499156263**

**43215** High-Sociable **3** **0.408656334834051**

**43215** High-Sociable **4** **0.226936114358204**

**43215** High-Sociable **5** **0.3**

**43215** High-Sociable **6** **0.267394839142419**

**43215** High-Sociable **7** **0.559910707166777**

**43219** Low-Sociable **1** **0.689202437604511**

**43219** Low-Sociable **2** **0.622896460095898**

**43219** Low-Sociable **3** **0.539907399467724**

**43219** Low-Sociable **4** **0.483218377133983**

**43219** Low-Sociable **5** **0.543139024560011**

**43219** Low-Sociable **6** **0.78835271294009**

**43219** Low-Sociable **7** **0.693902010373223**

**43221** High-Sociable **1** **0.69462219947249**

**43221** High-Sociable **2** **0.793410360406266**

**43221** High-Sociable **3** **0.612372435695795**

**43221** High-Sociable **4** **0.561693866799345**

**43221** High-Sociable **5** **0.510881590977792**

**43221** High-Sociable **6** **0.178885438199983**

**43221** High-Sociable **7** **0.433589667773576**

**43292** High-Sociable **1** **0.716240183178799**

**43292** High-Sociable **2** **0.574891294072192**

**43292** High-Sociable **3** **0.219089023002066**

**43292** High-Sociable **4** **0.95577193932444**

**43292** High-Sociable **5** **0.679705818718657**

**43292** High-Sociable **6** **0.452769256906871**

**43292** High-Sociable **7** **0.602494813255683**

**43298** High-Sociable **1** **0.716589143093865**

**43298** High-Sociable **2** **0.559910707166777**

**43298** High-Sociable **3** **0.476969600708473**

**43298** High-Sociable **4** **0.462060602085917**

**43298** High-Sociable **5** **0.674907401055878**

**43298** High-Sociable **6** **0.563032906675978**

**43298** High-Sociable **7** **0.634822809924155**

**43300** High-Sociable **1** **0.893697939462769**

**43300** High-Sociable **2** **0.925973928898649**

**43300** High-Sociable **3** **0.860970290428189**

**43300** High-Sociable **4** **0.906723580260269**

**43300** High-Sociable **5** **0.712039324756716**

**43300** High-Sociable **6** **0.547265931700485**

**43300** High-Sociable **7** **0**

**43395** High-Sociable **1** **0.735866835779409**

**43395** High-Sociable **2** **0.386652298583624**

**43395** High-Sociable **3** **0.291547594742265**

**43395** High-Sociable **4** **0.241867732448957**

**43395** High-Sociable **5** **0.1**

**43395** High-Sociable **6** **0.191049731745428**

**43395** High-Sociable **7** **0.420713679359253**

**43442** Low-Sociable **1** **0.733825592358293**

**43442** Low-Sociable **2** **0.584380013347479**

**43442** Low-Sociable **3** **0.668206554891525**

**43442** Low-Sociable **4** **0.549090156531694**

**43442** Low-Sociable **5** **0.365376518128902**

**43442** Low-Sociable **6** **0.452216762183801**

**43442** Low-Sociable **7** **0.453872228716409**

**43449** High-Sociable **1** **0.753657747256671**

**43449** High-Sociable **2** **0.544518135602479**

**43449** High-Sociable **3** **0.313847096529504**

**43449** High-Sociable **4** **0.367423461417477**

**43449** High-Sociable **5** **0.237697286480094**

**43449** High-Sociable **6** **0.216794833886788**

**43449** High-Sociable **7** **0**

**43523** High-Sociable **1** **0.505470078244005**

**43523** High-Sociable **2** **0.268328157299975**

**43523** High-Sociable **3** **0.168819430161341**

**43523** High-Sociable **4** **0.305777697028413**

**43523** High-Sociable **5** **0.168819430161341**

**43523** High-Sociable **6** **0.263628526529281**

**43523** High-Sociable **7** **0.271108834234519**

**43580** Low-Sociable **1** **0.790569415042095**

**43580** Low-Sociable **2** **0.483735464897913**

**43580** Low-Sociable **3** **0.586515131944607**

**43580** Low-Sociable **4** **0.248997991959775**

**43580** Low-Sociable **5** **0.389871773792359**

**43580** Low-Sociable **6** **0.216794833886788**

**43580** Low-Sociable **7** **0.496487663492256**

**43581** Low-Sociable **1** **0.702851335632223**

**43581** Low-Sociable **2** **0.257875939164553**

**43581** Low-Sociable **3** **0.208566536146142**

**43581** Low-Sociable **4** **0.787718223732319**

**43581** Low-Sociable **5** **0.780064099930256**

**43581** Low-Sociable **6** **0.777495980697007**

**43581** Low-Sociable **7** **0.587792480387424**

**43637** High-Sociable **1** **0.352408090145502**

**43637** High-Sociable **2** **0.555877684387492**

**43637** High-Sociable **3** **0.366742416417845**

**43637** High-Sociable **4** **0.4**

**43637** High-Sociable **5** **0.368103246386119**

**43637** High-Sociable **6** **0.201246117974981**

**43637** High-Sociable **7** **0.220227155455452**

**43675** High-Sociable **1** **0.854762583411324**

**43675** High-Sociable **2** **0.545893762558247**

**43675** High-Sociable **3** **0.576194411635517**

**43675** High-Sociable **4** **0.864002314811714**

**43675** High-Sociable **5** **0.677126280689208**

**43675** High-Sociable **6** **0.737902432574931**

**43675** High-Sociable **7** **0.616441400296898**

**43746** High-Sociable **1** **0.87091905479212**

**43746** High-Sociable **2** **0.399374510954317**

**43746** High-Sociable **3** **0.571839138219832**

**43746** High-Sociable **4** **0.493534767772241**

**43746** High-Sociable **5** **0.402492235949962**

**43746** High-Sociable **6** **0.29748949561287**

**43746** High-Sociable **7** **0.236643191323985**

**43854** High-Sociable **1** **0.634034699365894**

**43854** High-Sociable **2** **0.590338885725818**

**43854** High-Sociable **3** **0.659545297913646**

**43854** High-Sociable **4** **0.739256383131049**

**43854** High-Sociable **5** **0.271108834234519**

**43854** High-Sociable **6** **0.406201920231798**

**43854** High-Sociable **7** **0.33391615714128**

;

**RUN**;

**PROC** **MIXED** ASYCOV NOBOUND DATA=PL_task_repeated_measures_forma ALPHA=**0.025**;

CLASS ANIMID SociabilityText Session;

MODEL sqrt_DurFamil_ToT = SociabilityText Session Session*SociabilityText/ SOLUTION DDFM=KENWARDROGER;

RANDOM ANIMID(SociabilityText ) / SOLUTION ;

LSmeans SociabilityText;

**RUN**;

## Preference for Novel Faces in Recognition Trials

**DATA** PL_task_repeated_measures_forma; INPUT ANIMID SociabilityText &$16. Session Duration_ProbeMeanofAllTimeOnTar; Lines;

**40737** Low-Sociable **1** **0.510204082**

**40737** Low-Sociable **2** **0.365740741**

**40737** Low-Sociable **3** **0.899117276**

**40737** Low-Sociable **4** **0.2694702**

**40737** Low-Sociable **5** **0**

**40737** Low-Sociable **6** **0.195294118**

**40737** Low-Sociable **7** **1**

**41470** Low-Sociable **1** **0.516483516**

**41470** Low-Sociable **2** **0.37826087**

**41470** Low-Sociable **3** **0**

**41470** Low-Sociable **4** **0**

**41470** Low-Sociable **5** **0.376518219**

**41470** Low-Sociable **6** **0.746791132**

**41470** Low-Sociable **7** **0.122641509**

**41475** High-Sociable **1** **0.415**

**41475** High-Sociable **2** **0.61281337**

**41475** High-Sociable **3** **.**

**41475** High-Sociable **4** **0.14**

**41475** High-Sociable **5** **.**

**41475** High-Sociable **6** **.**

**41475** High-Sociable **7** **0**

**41518** High-Sociable **1** **0.201877934**

**41518** High-Sociable **2** **0.410187668**

**41518** High-Sociable **3** **0.327586207**

**41518** High-Sociable **4** **0.639468691**

**41518** High-Sociable **5** **0.38330494**

**41518** High-Sociable **6** **0.784444444**

**41518** High-Sociable **7** **0.482566248**

**41520** Low-Sociable **1** **0.10496614**

**41520** Low-Sociable **2** **0.46**

**41520** Low-Sociable **3** **1**

**41520** Low-Sociable **4** **1**

**41520** Low-Sociable **5** **0**

**41520** Low-Sociable **6** **0.368421053**

**41520** Low-Sociable **7** **0**

**41556** High-Sociable **1** **0.547826087**

**41556** High-Sociable **2** **0.882022472**

**41556** High-Sociable **3** **0.607038123**

**41556** High-Sociable **4** **0.550898204**

**41556** High-Sociable **5** **0.615858969**

**41556** High-Sociable **6** **0.525502318**

**41556** High-Sociable **7** **0.781893004**

**41569** Low-Sociable **1** **0.697247706**

**41569** Low-Sociable **2** **0.439252336**

**41569** Low-Sociable **3** **0.5**

**41569** Low-Sociable **4** **0**

**41569** Low-Sociable **5** **.**

**41569** Low-Sociable **6** **1**

**41569** Low-Sociable **7** **.**

**41659** Low-Sociable **1** **0.579695431**

**41659** Low-Sociable **2** **0.587646077**

**41659** Low-Sociable **3** **0.879251701**

**41659** Low-Sociable **4** **0.397486498**

**41659** Low-Sociable **5** **0.677173913**

**41659** Low-Sociable **6** **0.576253838**

**41659** Low-Sociable **7** **0.674496644**

**41662** Low-Sociable **1** **0.67357513**

**41662** Low-Sociable **2** **0.727586207**

**41662** Low-Sociable **3** **.**

**41662** Low-Sociable **4** **0**

**41662** Low-Sociable **5** **0.204347826**

**41662** Low-Sociable **6** **1**

**41662** Low-Sociable **7** **.**

**42039** Low-Sociable **1** **0.648854962**

**42039** Low-Sociable **2** **0.634241245**

**42039** Low-Sociable **3** **0.801512287**

**42039** Low-Sociable **4** **0.648648649**

**42039** Low-Sociable **5** **0**

**42039** Low-Sociable **6** **0**

**42039** Low-Sociable **7** **0.374203822**

**42127** High-Sociable **1** **0.701086957**

**42127** High-Sociable **2** **0.70846395**

**42127** High-Sociable **3** **0.816666667**

**42127** High-Sociable **4** **0.563439065**

**42127** High-Sociable **5** **0.554532578**

**42127** High-Sociable **6** **0.795683453**

**42127** High-Sociable **7** **0.692375887**

**42156** High-Sociable **1** **0.469548134**

**42156** High-Sociable **2** **0.709443099**

**42156** High-Sociable **3** **0.906**

**42156** High-Sociable **4** **0.118881119**

**42156** High-Sociable **5** **0**

**42156** High-Sociable **6** **0.748091603**

**42156** High-Sociable **7** **0.392255892**

**42183** Low-Sociable **1** **0.65391015**

**42183** Low-Sociable **2** **0.943548387**

**42183** Low-Sociable **3** **0.374531835**

**42183** Low-Sociable **4** **0.55704698**

**42183** Low-Sociable **5** **0.456896552**

**42183** Low-Sociable **6** **0.459459459**

**42183** Low-Sociable **7** **1**

**42283** Low-Sociable **1** **0.690451235**

**42283** Low-Sociable **2** **0.242290749**

**42283** Low-Sociable **3** **0.791013584**

**42283** Low-Sociable **4** **0.202360877**

**42283** Low-Sociable **5** **0.686695279**

**42283** Low-Sociable **6** **0.974248927**

**42283** Low-Sociable **7** **0.776859504**

**42299** Low-Sociable **1** **0.822888283**

**42299** Low-Sociable **2** **0**

**42299** Low-Sociable **3** **0.881205674**

**42299** Low-Sociable **4** **0**

**42299** Low-Sociable **5** **.**

**42299** Low-Sociable **6** **1**

**42299** Low-Sociable **7** **0**

**42324** High-Sociable **1** **0.478253278**

**42324** High-Sociable **2** **0.607963247**

**42324** High-Sociable **3** **1**

**42324** High-Sociable **4** **0**

**42324** High-Sociable **5** **0.627640845**

**42324** High-Sociable **6** **0.119170984**

**42324** High-Sociable **7** **0.092013889**

**42333** High-Sociable **1** **0.708283313**

**42333** High-Sociable **2** **0.843223664**

**42333** High-Sociable **3** **0.74566474**

**42333** High-Sociable **4** **0.392380393**

**42333** High-Sociable **5** **0.17989418**

**42333** High-Sociable **6** **.**

**42333** High-Sociable **7** **1**

**42369** High-Sociable **1** **0.478747204**

**42369** High-Sociable **2** **0.593272171**

**42369** High-Sociable **3** **0.761491549**

**42369** High-Sociable **4** **0.92414665**

**42369** High-Sociable **5** **0.646464646**

**42369** High-Sociable **6** **0.508474576**

**42369** High-Sociable **7** **0.328070175**

**42371** Low-Sociable **1** **0**

**42371** Low-Sociable **2** **0**

**42371** Low-Sociable **3** **1**

**42371** Low-Sociable **4** **.**

**42371** Low-Sociable **5** **0**

**42371** Low-Sociable **6** **.**

**42371** Low-Sociable **7** **0**

**42373** Low-Sociable **1** **0.506493506**

**42373** Low-Sociable **2** **0**

**42373** Low-Sociable **3** **0.910662824**

**42373** Low-Sociable **4** **0.498583569**

**42373** Low-Sociable **5** **0.674242424**

**42373** Low-Sociable **6** **0**

**42373** Low-Sociable **7** **0.576576577**

**42396** Low-Sociable **1** **0.880829016**

**42396** Low-Sociable **2** **0.607594937**

**42396** Low-Sociable **3** **0.566037736**

**42396** Low-Sociable **4** **0.14084507**

**42396** Low-Sociable **5** **1**

**42396** Low-Sociable **6** **.**

**42396** Low-Sociable **7** **0.31147541**

**42399** High-Sociable **1** **0.616858238**

**42399** High-Sociable **2** **0.806615776**

**42399** High-Sociable **3** **0.340909091**

**42399** High-Sociable **4** **0**

**42399** High-Sociable **5** **0**

**42399** High-Sociable **6** **1**

**42399** High-Sociable **7** **0.763157895**

**42402** Low-Sociable **1** **0.407920792**

**42402** Low-Sociable **2** **0.145772595**

**42402** Low-Sociable **3** **1**

**42402** Low-Sociable **4** **0.300601202**

**42402** Low-Sociable **5** **0.533333333**

**42402** Low-Sociable **6** **0.410447761**

**42402** Low-Sociable **7** **0.597014925**

**42612** Low-Sociable **1** **0.520134228**

**42612** Low-Sociable **2** **0.827089337**

**42612** Low-Sociable **3** **0.55465587**

**42612** Low-Sociable **4** **0**

**42612** Low-Sociable **5** **0.386792453**

**42612** Low-Sociable **6** **0.241538462**

**42612** Low-Sociable **7** **0**

**42672** High-Sociable **1** **0.638297872**

**42672** High-Sociable **2** **0**

**42672** High-Sociable **3** **1**

**42672** High-Sociable **4** **1**

**42672** High-Sociable **5** **0.576131687**

**42672** High-Sociable **6** **0.357142857**

**42672** High-Sociable **7** **.**

**42694** Low-Sociable **1** **0.620689655**

**42694** Low-Sociable **2** **0.497354497**

**42694** Low-Sociable **3** **.**

**42694** Low-Sociable **4** **0.083591331**

**42694** Low-Sociable **5** **0.27756654**

**42694** Low-Sociable **6** **.**

**42694** Low-Sociable **7** **0**

**42705** Low-Sociable **1** **0.608024691**

**42705** Low-Sociable **2** **0.703208556**

**42705** Low-Sociable **3** **0.453168044**

**42705** Low-Sociable **4** **1**

**42705** Low-Sociable **5** **0.536697248**

**42705** Low-Sociable **6** **1**

**42705** Low-Sociable **7** **0.601851852**

**42717** Low-Sociable **1** **0.57826087**

**42717** Low-Sociable **2** **0**

**42717** Low-Sociable **3** **.**

**42717** Low-Sociable **4** **0**

**42717** Low-Sociable **5** **.**

**42717** Low-Sociable **6** **0.575757576**

**42717** Low-Sociable **7** **1**

**42723** High-Sociable **1** **0.644351464**

**42723** High-Sociable **2** **0**

**42723** High-Sociable **3** **.**

**42723** High-Sociable **4** **0.569811321**

**42723** High-Sociable **5** **0.477509202**

**42723** High-Sociable **6** **0.641463415**

**42723** High-Sociable **7** **0.661512027**

**42725** High-Sociable **1** **0.450729927**

**42725** High-Sociable **2** **0.860215054**

**42725** High-Sociable **3** **0**

**42725** High-Sociable **4** **1**

**42725** High-Sociable **5** **1**

**42725** High-Sociable **6** **0.864197531**

**42725** High-Sociable **7** **1**

**42858** Low-Sociable **1** **0.158940397**

**42858** Low-Sociable **2** **0.692946058**

**42858** Low-Sociable **3** **0.871173469**

**42858** Low-Sociable **4** **.**

**42858** Low-Sociable **5** **0.601005868**

**42858** Low-Sociable **6** **0**

**42858** Low-Sociable **7** **.**

**42979** High-Sociable **1** **0.566037736**

**42979** High-Sociable **2** **1**

**42979** High-Sociable **3** **0.408988764**

**42979** High-Sociable **4** **0.681288409**

**42979** High-Sociable **5** **0.754299754**

**42979** High-Sociable **6** **0.391176471**

**42979** High-Sociable **7** **0**

**43131** Low-Sociable **1** **0.245850622**

**43131** Low-Sociable **2** **0.772052421**

**43131** Low-Sociable **3** **0.834575261**

**43131** Low-Sociable **4** **0.7**

**43131** Low-Sociable **5** **0.657754011**

**43131** Low-Sociable **6** **0**

**43131** Low-Sociable **7** **0**

**43157** Low-Sociable **1** **0.645746165**

**43157** Low-Sociable **2** **0.892307692**

**43157** Low-Sociable **3** **0.588150289**

**43157** Low-Sociable **4** **0.375**

**43157** Low-Sociable **5** **0**

**43157** Low-Sociable **6** **0.416430595**

**43157** Low-Sociable **7** **0.488235294**

**43215** High-Sociable **1** **1**

**43215** High-Sociable **2** **0**

**43215** High-Sociable **3** **.**

**43215** High-Sociable **4** **0.603896104**

**43215** High-Sociable **5** **0.333333333**

**43215** High-Sociable **6** **1**

**43215** High-Sociable **7** **0**

**43219** Low-Sociable **1** **0.473684211**

**43219** Low-Sociable **2** **0.409090909**

**43219** Low-Sociable **3** **0.529411765**

**43219** Low-Sociable **4** **0**

**43219** Low-Sociable **5** **0.255439924**

**43219** Low-Sociable **6** **0.605790646**

**43219** Low-Sociable **7** **0.567375887**

**43221** High-Sociable **1** **0.746085011**

**43221** High-Sociable **2** **0.432806324**

**43221** High-Sociable **3** **0.75**

**43221** High-Sociable **4** **0.15052161**

**43221** High-Sociable **5** **0.570162482**

**43221** High-Sociable **6** **0.435986159**

**43221** High-Sociable **7** **0.608359133**

**43292** High-Sociable **1** **0.255319149**

**43292** High-Sociable **2** **0.413265306**

**43292** High-Sociable **3** **0.531179564**

**43292** High-Sociable **4** **0.662232077**

**43292** High-Sociable **5** **0.302267003**

**43292** High-Sociable **6** **1**

**43292** High-Sociable **7** **0.032786885**

**43298** High-Sociable **1** **0.733520337**

**43298** High-Sociable **2** **0.363507779**

**43298** High-Sociable **3** **0.703832753**

**43298** High-Sociable **4** **0.444444444**

**43298** High-Sociable **5** **1**

**43298** High-Sociable **6** **0**

**43298** High-Sociable **7** **0.2**

**43300** High-Sociable **1** **0.65895948**

**43300** High-Sociable **2** **0.537323944**

**43300** High-Sociable **3** **0.894706054**

**43300** High-Sociable **4** **0.306737589**

**43300** High-Sociable **5** **0.634462151**

**43300** High-Sociable **6** **0.368472088**

**43300** High-Sociable **7** **0.597235023**

**43395** High-Sociable **1** **0.607085346**

**43395** High-Sociable **2** **0.20979021**

**43395** High-Sociable **3** **1**

**43395** High-Sociable **4** **0**

**43395** High-Sociable **5** **0.263157895**

**43395** High-Sociable **6** **0.740740741**

**43395** High-Sociable **7** **0.893617021**

**43442** Low-Sociable **1** **0.430288462**

**43442** Low-Sociable **2** **0.69928401**

**43442** Low-Sociable **3** **0.320675105**

**43442** Low-Sociable **4** **0.540594059**

**43442** Low-Sociable **5** **0**

**43442** Low-Sociable **6** **0.288201161**

**43442** Low-Sociable **7** **1**

**43449** High-Sociable **1** **0.478087649**

**43449** High-Sociable **2** **0**

**43449** High-Sociable **3** **0.825581395**

**43449** High-Sociable **4** **0.325**

**43449** High-Sociable **5** **0**

**43449** High-Sociable **6** **0.759036145**

**43449** High-Sociable **7** **1**

**43523** High-Sociable **1** **0.724137931**

**43523** High-Sociable **2** **0.840255591**

**43523** High-Sociable **3** **0.701492537**

**43523** High-Sociable **4** **1**

**43523** High-Sociable **5** **0.123655914**

**43523** High-Sociable **6** **0.823076923**

**43523** High-Sociable **7** **0.381443299**

**43580** Low-Sociable **1** **0.569131833**

**43580** Low-Sociable **2** **0.146788991**

**43580** Low-Sociable **3** **0.7135506**

**43580** Low-Sociable **4** **0.698198198**

**43580** Low-Sociable **5** **0.721649485**

**43580** Low-Sociable **6** **0.939244663**

**43580** Low-Sociable **7** **0.613636364**

**43581** Low-Sociable **1** **0.75128866**

**43581** Low-Sociable **2** **0.349363508**

**43581** Low-Sociable **3** **0.668918919**

**43581** Low-Sociable **4** **0.166666667**

**43581** Low-Sociable **5** **.**

**43581** Low-Sociable **6** **0.566617862**

**43581** Low-Sociable **7** **0.694516971**

**43637** High-Sociable **1** **0.80875576**

**43637** High-Sociable **2** **0.351421189**

**43637** High-Sociable **3** **1**

**43637** High-Sociable **4** **1**

**43637** High-Sociable **5** **0.812206573**

**43637** High-Sociable **6** **0.128668172**

**43637** High-Sociable **7** **0.439868204**

**43675** High-Sociable **1** **0.718778077**

**43675** High-Sociable **2** **0.551616267**

**43675** High-Sociable **3** **0.803196803**

**43675** High-Sociable **4** **0.518754988**

**43675** High-Sociable **5** **0**

**43675** High-Sociable **6** **0.875**

**43675** High-Sociable **7** **0.514619883**

**43746** High-Sociable **1** **0.756756757**

**43746** High-Sociable **2** **0.923863636**

**43746** High-Sociable **3** **0.1875**

**43746** High-Sociable **4** **0.588235294**

**43746** High-Sociable **5** **1**

**43746** High-Sociable **6** **1**

**43746** High-Sociable **7** **0**

**43854** High-Sociable **1** **0.296150049**

**43854** High-Sociable **2** **0.665211063**

**43854** High-Sociable **3** **0.806763285**

**43854** High-Sociable **4** **0.322352941**

**43854** High-Sociable **5** **0.74382716**

**43854** High-Sociable **6** **1**

**43854** High-Sociable **7** **1**

;

**RUN**;

**PROC** **MIXED** ASYCOV NOBOUND DATA=PL_task_repeated_measures_forma ;

CLASS ANIMID SociabilityText Session;

MODEL Duration_ProbeMeanofAllTimeOnTar = SociabilityText Session Session*SociabilityText/ SOLUTION DDFM=KENWARDROGER;

RANDOM ANIMID(SociabilityText ) / SOLUTION ;

lsmeans SociabilityText/ CL ALPHA=**0.025** ;

**RUN**;

# Socially Appropriate Gaze Aversion Test

## Rate of Gaze Avert

**DATA** VP_task_repeated_measures_forma; INPUT ID SociabilityText &$16. OrderedSection &$16. SectionType &$16. sqrt_GazeAvert_RPM; Lines;

**40737** Low-Sociable **1**_NS1 NS **3.554093267**

**40737** Low-Sociable **2**_AG1 AG **3.464101615**

**40737** Low-Sociable **3**_NS2 NS **3.142199504**

**40737** Low-Sociable **4**_AG2 AG **3.508232077**

**40737** Low-Sociable **5**_NS3 NS **2.966479395**

**40737** Low-Sociable **6**_AG3 AG **3.048897284**

**40737** Low-Sociable **7**_NS4 NS **3.027650354**

**41470** Low-Sociable **1**_NS1 NS **2.294157339**

**41470** Low-Sociable **2**_AG1 AG **3.055050463**

**41470** Low-Sociable **3**_NS2 NS **4.357446703**

**41470** Low-Sociable **4**_AG2 AG **3.922322703**

**41470** Low-Sociable **5**_NS3 NS **4.195235393**

**41470** Low-Sociable **6**_AG3 AG **2.60010834**

**41470** Low-Sociable **7**_NS4 NS **4.183300133**

**41475** High-Sociable **1**_NS1 NS **2.51312345**

**41475** High-Sociable **2**_AG1 AG **3.741657387**

**41475** High-Sociable **3**_NS2 NS **3.798733966**

**41475** High-Sociable **4**_AG2 AG **3.77236918**

**41475** High-Sociable **5**_NS3 NS **3.687817783**

**41475** High-Sociable **6**_AG3 AG **2.757831359**

**41475** High-Sociable **7**_NS4 NS **3.535533906**

**41518** High-Sociable **1**_NS1 NS **2.71448357**

**41518** High-Sociable **2**_AG1 AG **4.163331999**

**41518** High-Sociable **3**_NS2 NS **4.773343705**

**41518** High-Sociable **4**_AG2 AG **4.251696494**

**41518** High-Sociable **5**_NS3 NS **4.472135955**

**41518** High-Sociable **6**_AG3 AG **3.790276663**

**41518** High-Sociable **7**_NS4 NS **4.915960401**

**41520** Low-Sociable **1**_NS1 NS **3.077935056**

**41520** Low-Sociable **2**_AG1 AG **3.366501646**

**41520** Low-Sociable **3**_NS2 NS **3.798733966**

**41520** Low-Sociable **4**_AG2 AG **3.222516933**

**41520** Low-Sociable **5**_NS3 NS **3.794733192**

**41520** Low-Sociable **6**_AG3 AG **2.757831359**

**41520** Low-Sociable **7**_NS4 NS **4.472135955**

**41566** High-Sociable **1**_NS1 NS **2.51312345**

**41566** High-Sociable **2**_AG1 AG **2.943920289**

**41566** High-Sociable **3**_NS2 NS **3.375263703**

**41566** High-Sociable **4**_AG2 AG **2.55704156**

**41566** High-Sociable **5**_NS3 NS **2.683281573**

**41566** High-Sociable **6**_AG3 AG **2.60010834**

**41566** High-Sociable **7**_NS4 NS **3.763863264**

**41569** Low-Sociable **1**_NS1 NS **3.244428423**

**41569** Low-Sociable **2**_AG1 AG **3.915780041**

**41569** Low-Sociable **3**_NS2 NS **3.798733966**

**41569** Low-Sociable **4**_AG2 AG **3.971049077**

**41569** Low-Sociable **5**_NS3 NS **4.647580015**

**41569** Low-Sociable **6**_AG3 AG **2.60010834**

**41569** Low-Sociable **7**_NS4 NS **3.872983346**

**41659** Low-Sociable **1**_NS1 NS **4.103913408**

**41659** Low-Sociable **2**_AG1 AG **4.163331999**

**41659** Low-Sociable **3**_NS2 NS **3.697416135**

**41659** Low-Sociable **4**_AG2 AG **3.222516933**

**41659** Low-Sociable **5**_NS3 NS **4.38178046**

**41659** Low-Sociable **6**_AG3 AG **3.184469355**

**41659** Low-Sociable **7**_NS4 NS **4.830458915**

**41662** Low-Sociable **1**_NS1 NS **3.244428423**

**41662** Low-Sociable **2**_AG1 AG **2.581988897**

**41662** Low-Sociable **3**_NS2 NS **2.755891273**

**41662** Low-Sociable **4**_AG2 AG **2.55704156**

**41662** Low-Sociable **5**_NS3 NS **3.098386677**

**41662** Low-Sociable **6**_AG3 AG **3.439620025**

**41662** Low-Sociable **7**_NS4 NS **3.415650255**

**42039** Low-Sociable **1**_NS1 NS **3.244428423**

**42039** Low-Sociable **2**_AG1 AG **2.708012802**

**42039** Low-Sociable **3**_NS2 NS **3.798733966**

**42039** Low-Sociable **4**_AG2 AG **3.396831102**

**42039** Low-Sociable **5**_NS3 NS **3.346640106**

**42039** Low-Sociable **6**_AG3 AG **3.90016251**

**42039** Low-Sociable **7**_NS4 NS **3.651483717**

**42127** High-Sociable **1**_NS1 NS **2.71448357**

**42127** High-Sociable **2**_AG1 AG **4.242640687**

**42127** High-Sociable **3**_NS2 NS **4.357446703**

**42127** High-Sociable **4**_AG2 AG **4.113766756**

**42127** High-Sociable **5**_NS3 NS **4.195235393**

**42127** High-Sociable **6**_AG3 AG **3.90016251**

**42127** High-Sociable **7**_NS4 NS **4.564354646**

**42156** High-Sociable **1**_NS1 NS **3.402785237**

**42156** High-Sociable **2**_AG1 AG **4.546060566**

**42156** High-Sociable **3**_NS2 NS **5.228936044**

**42156** High-Sociable **4**_AG2 AG **5.038314737**

**42156** High-Sociable **5**_NS3 NS **4.898979486**

**42156** High-Sociable **6**_AG3 AG **5.118318386**

**42156** High-Sociable **7**_NS4 NS **5.627314339**

**42183** Low-Sociable **1**_NS1 NS **3.402785237**

**42183** Low-Sociable **2**_AG1 AG **4.163331999**

**42183** Low-Sociable **3**_NS2 NS **3.897418815**

**42183** Low-Sociable **4**_AG2 AG **4.019184762**

**42183** Low-Sociable **5**_NS3 NS **4.098780306**

**42183** Low-Sociable **6**_AG3 AG **3.184469355**

**42183** Low-Sociable **7**_NS4 NS **3.872983346**

**42283** Low-Sociable **1**_NS1 NS **4.103913408**

**42283** Low-Sociable **2**_AG1 AG **3.559026084**

**42283** Low-Sociable **3**_NS2 NS **3.798733966**

**42283** Low-Sociable **4**_AG2 AG **3.562626516**

**42283** Low-Sociable **5**_NS3 NS **3.346640106**

**42283** Low-Sociable **6**_AG3 AG **4.311791889**

**42283** Low-Sociable **7**_NS4 NS **3.651483717**

**42299** Low-Sociable **1**_NS1 NS **3.244428423**

**42299** Low-Sociable **2**_AG1 AG **4.320493799**

**42299** Low-Sociable **3**_NS2 NS **3.798733966**

**42299** Low-Sociable **4**_AG2 AG **4.428925899**

**42299** Low-Sociable **5**_NS3 NS **3.794733192**

**42299** Low-Sociable **6**_AG3 AG **4.864357289**

**42299** Low-Sociable **7**_NS4 NS **4.281744193**

**42324** High-Sociable **1**_NS1 NS **3.554093267**

**42324** High-Sociable **2**_AG1 AG **3.651483717**

**42324** High-Sociable **3**_NS2 NS **3.260814529**

**42324** High-Sociable **4**_AG2 AG **3.508232077**

**42324** High-Sociable **5**_NS3 NS **3.464101615**

**42324** High-Sociable **6**_AG3 AG **3.790276663**

**42324** High-Sociable **7**_NS4 NS **3.415650255**

**42333** High-Sociable **1**_NS1 NS **4.230217115**

**42333** High-Sociable **2**_AG1 AG **3.741657387**

**42333** High-Sociable **3**_NS2 NS **4.528391449**

**42333** High-Sociable **4**_AG2 AG **4.803844614**

**42333** High-Sociable **5**_NS3 NS **4.732863826**

**42333** High-Sociable **6**_AG3 AG **4.864357289**

**42333** High-Sociable **7**_NS4 NS **4.915960401**

**42369** High-Sociable **1**_NS1 NS **3.83885948**

**42369** High-Sociable **2**_AG1 AG **3.915780041**

**42369** High-Sociable **3**_NS2 NS **4.611488131**

**42369** High-Sociable **4**_AG2 AG **4.428925899**

**42369** High-Sociable **5**_NS3 NS **5.865151319**

**42369** High-Sociable **6**_AG3 AG **4.408698189**

**42369** High-Sociable **7**_NS4 NS **5.477225575**

**42371** Low-Sociable **1**_NS1 NS **3.244428423**

**42371** Low-Sociable **2**_AG1 AG **2.943920289**

**42371** Low-Sociable **3**_NS2 NS **3.593242603**

**42371** Low-Sociable **4**_AG2 AG **2.320477404**

**42371** Low-Sociable **5**_NS3 NS **3.464101615**

**42371** Low-Sociable **6**_AG3 AG **2.907009499**

**42371** Low-Sociable **7**_NS4 NS **3.535533906**

**42373** Low-Sociable **1**_NS1 NS **3.402785237**

**42373** Low-Sociable **2**_AG1 AG **3.464101615**

**42373** Low-Sociable **3**_NS2 NS **2.305744066**

**42373** Low-Sociable **4**_AG2 AG **2.974248451**

**42373** Low-Sociable **5**_NS3 NS **2.19089023**

**42373** Low-Sociable **6**_AG3 AG **3.314500791**

**42373** Low-Sociable **7**_NS4 NS **2.415229458**

**42396** Low-Sociable **1**_NS1 NS **3.699217556**

**42396** Low-Sociable **2**_AG1 AG **2.943920289**

**42396** Low-Sociable **3**_NS2 NS **3.485957363**

**42396** Low-Sociable **4**_AG2 AG **4.296689244**

**42396** Low-Sociable **5**_NS3 NS **4**

**42396** Low-Sociable **6**_AG3 AG **4.311791889**

**42396** Low-Sociable **7**_NS4 NS **4.472135955**

**42399** High-Sociable **1**_NS1 NS **3.699217556**

**42399** High-Sociable **2**_AG1 AG **3.464101615**

**42399** High-Sociable **3**_NS2 NS **3.018927633**

**42399** High-Sociable **4**_AG2 AG **4.599331055**

**42399** High-Sociable **5**_NS3 NS **4.195235393**

**42399** High-Sociable **6**_AG3 AG **4.212656985**

**42399** High-Sociable **7**_NS4 NS **4.281744193**

**42402** Low-Sociable **1**_NS1 NS **2.71448357**

**42402** Low-Sociable **2**_AG1 AG **2.943920289**

**42402** Low-Sociable **3**_NS2 NS **3.897418815**

**42402** Low-Sociable **4**_AG2 AG **3.452980875**

**42402** Low-Sociable **5**_NS3 NS **4.647580015**

**42402** Low-Sociable **6**_AG3 AG **2.757831359**

**42402** Low-Sociable **7**_NS4 NS **4.281744193**

**42612** Low-Sociable **1**_NS1 NS **3.244428423**

**42612** Low-Sociable **2**_AG1 AG **2.943920289**

**42612** Low-Sociable **3**_NS2 NS **3.142199504**

**42612** Low-Sociable **4**_AG2 AG **2.401922307**

**42612** Low-Sociable **5**_NS3 NS **3.898717738**

**42612** Low-Sociable **6**_AG3 AG **2.251759875**

**42612** Low-Sociable **7**_NS4 NS **3.535533906**

**42672** High-Sociable **1**_NS1 NS **3.554093267**

**42672** High-Sociable **2**_AG1 AG **4.472135955**

**42672** High-Sociable **3**_NS2 NS **3.798733966**

**42672** High-Sociable **4**_AG2 AG **4.557327152**

**42672** High-Sociable **5**_NS3 NS **3.794733192**

**42672** High-Sociable **6**_AG3 AG **5.438516782**

**42672** High-Sociable **7**_NS4 NS **4.082482905**

**42694** Low-Sociable **1**_NS1 NS **3.699217556**

**42694** Low-Sociable **2**_AG1 AG **3.265986324**

**42694** Low-Sociable **3**_NS2 NS **2.305744066**

**42694** Low-Sociable **4**_AG2 AG **3.339737438**

**42694** Low-Sociable **5**_NS3 NS **3.098386677**

**42694** Low-Sociable **6**_AG3 AG **3.439620025**

**42694** Low-Sociable **7**_NS4 NS **3.415650255**

**42705** Low-Sociable **1**_NS1 NS **3.402785237**

**42705** Low-Sociable **2**_AG1 AG **3.366501646**

**42705** Low-Sociable **3**_NS2 NS **2.890403152**

**42705** Low-Sociable **4**_AG2 AG **2.8419928**

**42705** Low-Sociable **5**_NS3 NS **3.098386677**

**42705** Low-Sociable **6**_AG3 AG **1.592234677**

**42705** Low-Sociable **7**_NS4 NS **2.886751346**

**42717** Low-Sociable **1**_NS1 NS **4.103913408**

**42717** Low-Sociable **2**_AG1 AG **4.618802154**

**42717** Low-Sociable **3**_NS2 NS **4.611488131**

**42717** Low-Sociable **4**_AG2 AG **5.11408312**

**42717** Low-Sociable **5**_NS3 NS **4.732863826**

**42717** Low-Sociable **6**_AG3 AG **5.438516782**

**42717** Low-Sociable **7**_NS4 NS **5.244044241**

**42723** High-Sociable **1**_NS1 NS **2.51312345**

**42723** High-Sociable **2**_AG1 AG **3.265986324**

**42723** High-Sociable **3**_NS2 NS **3.993665871**

**42723** High-Sociable **4**_AG2 AG **3.616202853**

**42723** High-Sociable **5**_NS3 NS **4**

**42723** High-Sociable **6**_AG3 AG **3.90016251**

**42723** High-Sociable **7**_NS4 NS **4.377975179**

**42725** High-Sociable **1**_NS1 NS **3.699217556**

**42725** High-Sociable **2**_AG1 AG **3.055050463**

**42725** High-Sociable **3**_NS2 NS **4.269408402**

**42725** High-Sociable **4**_AG2 AG **3.668996929**

**42725** High-Sociable **5**_NS3 NS **4**

**42725** High-Sociable **6**_AG3 AG **4.111132259**

**42725** High-Sociable **7**_NS4 NS **4.377975179**

**42858** Low-Sociable **1**_NS1 NS **3.554093267**

**42858** Low-Sociable **2**_AG1 AG **3.741657387**

**42858** Low-Sociable **3**_NS2 NS **3.485957363**

**42858** Low-Sociable **4**_AG2 AG **2.908872369**

**42858** Low-Sociable **5**_NS3 NS **3.794733192**

**42858** Low-Sociable **6**_AG3 AG **3.439620025**

**42858** Low-Sociable **7**_NS4 NS **3.979112129**

**42979** High-Sociable **1**_NS1 NS **3.554093267**

**42979** High-Sociable **2**_AG1 AG **4.082482905**

**42979** High-Sociable **3**_NS2 NS **2.46494409**

**42979** High-Sociable **4**_AG2 AG **3.452980875**

**42979** High-Sociable **5**_NS3 NS **3.464101615**

**42979** High-Sociable **6**_AG3 AG **3.439620025**

**42979** High-Sociable **7**_NS4 NS **3.027650354**

**43131** Low-Sociable **1**_NS1 NS **2.294157339**

**43131** Low-Sociable **2**_AG1 AG **2.708012802**

**43131** Low-Sociable **3**_NS2 NS **3.993665871**

**43131** Low-Sociable **4**_AG2 AG **3.616202853**

**43131** Low-Sociable **5**_NS3 NS **3.898717738**

**43131** Low-Sociable **6**_AG3 AG **4.007036065**

**43131** Low-Sociable **7**_NS4 NS **4.183300133**

**43157** Low-Sociable **1**_NS1 NS **3.244428423**

**43157** Low-Sociable **2**_AG1 AG **2.828427125**

**43157** Low-Sociable **3**_NS2 NS **3.485957363**

**43157** Low-Sociable **4**_AG2 AG **3.616202853**

**43157** Low-Sociable **5**_NS3 NS **4.098780306**

**43157** Low-Sociable **6**_AG3 AG **3.560344975**

**43157** Low-Sociable **7**_NS4 NS **4.281744193**

**43215** High-Sociable **1**_NS1 NS **3.699217556**

**43215** High-Sociable **2**_AG1 AG **4.396968653**

**43215** High-Sociable **3**_NS2 NS **4.087647338**

**43215** High-Sociable **4**_AG2 AG **4.251696494**

**43215** High-Sociable **5**_NS3 NS **3.794733192**

**43215** High-Sociable **6**_AG3 AG **3.677108478**

**43215** High-Sociable **7**_NS4 NS **4.377975179**

**43219** Low-Sociable **1**_NS1 NS **3.973597071**

**43219** Low-Sociable **2**_AG1 AG **4.163331999**

**43219** Low-Sociable **3**_NS2 NS **5.228936044**

**43219** Low-Sociable **4**_AG2 AG **4.599331055**

**43219** Low-Sociable **5**_NS3 NS **4.979959839**

**43219** Low-Sociable **6**_AG3 AG **4.50351975**

**43219** Low-Sociable **7**_NS4 NS **5.244044241**

**43221** High-Sociable **1**_NS1 NS **3.554093267**

**43221** High-Sociable **2**_AG1 AG **4.618802154**

**43221** High-Sociable **3**_NS2 NS **5.372221095**

**43221** High-Sociable **4**_AG2 AG **4.922475925**

**43221** High-Sociable **5**_NS3 NS **4.898979486**

**43221** High-Sociable **6**_AG3 AG **5.20021668**

**43221** High-Sociable **7**_NS4 NS **5.163977795**

**43292** High-Sociable **1**_NS1 NS **2.901905**

**43292** High-Sociable **2**_AG1 AG **4.082482905**

**43292** High-Sociable **3**_NS2 NS **3.697416135**

**43292** High-Sociable **4**_AG2 AG **4.640954809**

**43292** High-Sociable **5**_NS3 NS **3.898717738**

**43292** High-Sociable **6**_AG3 AG **4.111132259**

**43292** High-Sociable **7**_NS4 NS **3.535533906**

**43298** High-Sociable **1**_NS1 NS **3.973597071**

**43298** High-Sociable **2**_AG1 AG **3.829708431**

**43298** High-Sociable **3**_NS2 NS **4.087647338**

**43298** High-Sociable **4**_AG2 AG **3.396831102**

**43298** High-Sociable **5**_NS3 NS **2.683281573**

**43298** High-Sociable **6**_AG3 AG **2.907009499**

**43298** High-Sociable **7**_NS4 NS **3.027650354**

**43300** High-Sociable **1**_NS1 NS **2.71448357**

**43300** High-Sociable **2**_AG1 AG **3.366501646**

**43300** High-Sociable **3**_NS2 NS **3.375263703**

**43300** High-Sociable **4**_AG2 AG **3.616202853**

**43300** High-Sociable **5**_NS3 NS **3.687817783**

**43300** High-Sociable **6**_AG3 AG **3.560344975**

**43300** High-Sociable **7**_NS4 NS **3.651483717**

**43395** High-Sociable **1**_NS1 NS **3.554093267**

**43395** High-Sociable **2**_AG1 AG **3.915780041**

**43395** High-Sociable **3**_NS2 NS **3.018927633**

**43395** High-Sociable **4**_AG2 AG **3.508232077**

**43395** High-Sociable **5**_NS3 NS **1.788854382**

**43395** High-Sociable **6**_AG3 AG **3.677108478**

**43395** High-Sociable **7**_NS4 NS **3.535533906**

**43442** Low-Sociable **1**_NS1 NS **4.230217115**

**43442** Low-Sociable **2**_AG1 AG **3.915780041**

**43442** Low-Sociable **3**_NS2 NS **4.357446703**

**43442** Low-Sociable **4**_AG2 AG **3.721042038**

**43442** Low-Sociable **5**_NS3 NS **3.687817783**

**43442** Low-Sociable **6**_AG3 AG **4.111132259**

**43442** Low-Sociable **7**_NS4 NS **3.535533906**

**43449** High-Sociable **1**_NS1 NS **3.244428423**

**43449** High-Sociable **2**_AG1 AG **3.464101615**

**43449** High-Sociable **3**_NS2 NS **3.142199504**

**43449** High-Sociable **4**_AG2 AG **3.339737438**

**43449** High-Sociable **5**_NS3 NS **1.788854382**

**43449** High-Sociable **6**_AG3 AG **3.677108478**

**43449** High-Sociable **7**_NS4 NS **3.291402943**

**43523** High-Sociable **1**_NS1 NS **3.244428423**

**43523** High-Sociable **2**_AG1 AG **4.163331999**

**43523** High-Sociable **3**_NS2 NS **3.697416135**

**43523** High-Sociable **4**_AG2 AG **4.113766756**

**43523** High-Sociable **5**_NS3 NS **4.195235393**

**43523** High-Sociable **6**_AG3 AG **3.677108478**

**43523** High-Sociable **7**_NS4 NS **3.979112129**

**43580** Low-Sociable **1**_NS1 NS **3.554093267**

**43580** Low-Sociable **2**_AG1 AG **3.559026084**

**43580** Low-Sociable **3**_NS2 NS **3.593242603**

**43580** Low-Sociable **4**_AG2 AG **3.77236918**

**43580** Low-Sociable **5**_NS3 NS **3.898717738**

**43580** Low-Sociable **6**_AG3 AG **3.90016251**

**43580** Low-Sociable **7**_NS4 NS **3.872983346**

**43581** Low-Sociable **1**_NS1 NS **3.077935056**

**43581** Low-Sociable **2**_AG1 AG **3.741657387**

**43581** Low-Sociable **3**_NS2 NS **3.485957363**

**43581** Low-Sociable **4**_AG2 AG **3.616202853**

**43581** Low-Sociable **5**_NS3 NS **4.098780306**

**43581** Low-Sociable **6**_AG3 AG **4.212656985**

**43581** Low-Sociable **7**_NS4 NS **4.281744193**

**43637** High-Sociable **1**_NS1 NS **3.244428423**

**43637** High-Sociable **2**_AG1 AG **3.651483717**

**43637** High-Sociable **3**_NS2 NS **3.018927633**

**43637** High-Sociable **4**_AG2 AG **3.872983346**

**43637** High-Sociable **5**_NS3 NS **3.224903099**

**43637** High-Sociable **6**_AG3 AG **3.677108478**

**43637** High-Sociable **7**_NS4 NS **4.183300133**

**43675** High-Sociable **1**_NS1 NS **2.71448357**

**43675** High-Sociable **2**_AG1 AG **3.741657387**

**43675** High-Sociable **3**_NS2 NS **3.993665871**

**43675** High-Sociable **4**_AG2 AG **3.971049077**

**43675** High-Sociable **5**_NS3 NS **3.898717738**

**43675** High-Sociable **6**_AG3 AG **3.314500791**

**43675** High-Sociable **7**_NS4 NS **4.183300133**

**43746** High-Sociable **1**_NS1 NS **3.244428423**

**43746** High-Sociable **2**_AG1 AG **4**

**43746** High-Sociable **3**_NS2 NS **4.087647338**

**43746** High-Sociable **4**_AG2 AG **4.472135955**

**43746** High-Sociable **5**_NS3 NS **3.794733192**

**43746** High-Sociable **6**_AG3 AG **4.50351975**

**43746** High-Sociable **7**_NS4 NS **4.377975179**

**43854** High-Sociable **1**_NS1 NS **3.244428423**

**43854** High-Sociable **2**_AG1 AG **3.464101615**

**43854** High-Sociable **3**_NS2 NS **3.697416135**

**43854** High-Sociable **4**_AG2 AG **4.428925899**

**43854** High-Sociable **5**_NS3 NS **4.732863826**

**43854** High-Sociable **6**_AG3 AG **5.118318386**

**43854** High-Sociable **7**_NS4 NS **4.472135955**

;

**RUN**;

**PROC** **MIXED** ASYCOV NOBOUND DATA=VP_task_repeated_measures_forma ALPHA=**0.05**;

CLASS ID SociabilityText OrderedSection SectionType;

MODEL sqrt_GazeAvert_RPM = SociabilityText OrderedSection(SectionType ) SectionType OrderedSection*SociabilityText(SectionType ) SectionType*SociabilityText/ SOLUTION DDFM=KENWARDROGER;

RANDOM ID(SociabilityText ) ID*SectionType(SociabilityText ) / SOLUTION ;

lsmeans SectionType*SociabilityText / slice=sectiontype slice=sociabilitytext ;

**RUN**;

## Rate of Looking

**DATA** VP_task_repeated_measures_forma; INPUT ID SociabilityText &$16. OrderedSection &$16. SectionType &$16. Look_RPM; Lines;

**40737** Low-Sociable **1**_NS1 NS **9.473684211**

**40737** Low-Sociable **2**_AG1 AG **10.66666667**

**40737** Low-Sociable **3**_NS2 NS **9.873417722**

**40737** Low-Sociable **4**_AG2 AG **10.76923077**

**40737** Low-Sociable **5**_NS3 NS **6.4**

**40737** Low-Sociable **6**_AG3 AG **8.450704225**

**40737** Low-Sociable **7**_NS4 NS **6.666666667**

**41470** Low-Sociable **1**_NS1 NS **7.368421053**

**41470** Low-Sociable **2**_AG1 AG **11.33333333**

**41470** Low-Sociable **3**_NS2 NS **18.2278481**

**41470** Low-Sociable **4**_AG2 AG **17.69230769**

**41470** Low-Sociable **5**_NS3 NS **20**

**41470** Low-Sociable **6**_AG3 AG **10.14084507**

**41470** Low-Sociable **7**_NS4 NS **19.16666667**

**41475** High-Sociable **1**_NS1 NS **6.315789474**

**41475** High-Sociable **2**_AG1 AG **12.66666667**

**41475** High-Sociable **3**_NS2 NS **11.39240506**

**41475** High-Sociable **4**_AG2 AG **17.30769231**

**41475** High-Sociable **5**_NS3 NS **17.6**

**41475** High-Sociable **6**_AG3 AG **17.74647887**

**41475** High-Sociable **7**_NS4 NS **17.5**

**41518** High-Sociable **1**_NS1 NS **8.421052632**

**41518** High-Sociable **2**_AG1 AG **18.66666667**

**41518** High-Sociable **3**_NS2 NS **21.26582278**

**41518** High-Sociable **4**_AG2 AG **18.46153846**

**41518** High-Sociable **5**_NS3 NS **22.4**

**41518** High-Sociable **6**_AG3 AG **16.05633803**

**41518** High-Sociable **7**_NS4 NS **25**

**41520** Low-Sociable **1**_NS1 NS **9.473684211**

**41520** Low-Sociable **2**_AG1 AG **13.33333333**

**41520** Low-Sociable **3**_NS2 NS **15.18987342**

**41520** Low-Sociable **4**_AG2 AG **13.07692308**

**41520** Low-Sociable **5**_NS3 NS **16**

**41520** Low-Sociable **6**_AG3 AG **11.83098592**

**41520** Low-Sociable **7**_NS4 NS **19.16666667**

**41566** High-Sociable **1**_NS1 NS **6.315789474**

**41566** High-Sociable **2**_AG1 AG **10.66666667**

**41566** High-Sociable **3**_NS2 NS **9.873417722**

**41566** High-Sociable **4**_AG2 AG **9.230769231**

**41566** High-Sociable **5**_NS3 NS **9.6**

**41566** High-Sociable **6**_AG3 AG **10.98591549**

**41566** High-Sociable **7**_NS4 NS **15.83333333**

**41569** Low-Sociable **1**_NS1 NS **8.421052632**

**41569** Low-Sociable **2**_AG1 AG **13.33333333**

**41569** Low-Sociable **3**_NS2 NS **11.39240506**

**41569** Low-Sociable **4**_AG2 AG **15.76923077**

**41569** Low-Sociable **5**_NS3 NS **15.2**

**41569** Low-Sociable **6**_AG3 AG **6.76056338**

**41569** Low-Sociable **7**_NS4 NS **13.33333333**

**41659** Low-Sociable **1**_NS1 NS **14.73684211**

**41659** Low-Sociable **2**_AG1 AG **16.66666667**

**41659** Low-Sociable **3**_NS2 NS **15.18987342**

**41659** Low-Sociable **4**_AG2 AG **12.30769231**

**41659** Low-Sociable **5**_NS3 NS **18.4**

**41659** Low-Sociable **6**_AG3 AG **8.450704225**

**41659** Low-Sociable **7**_NS4 NS **23.33333333**

**41662** Low-Sociable **1**_NS1 NS **11.57894737**

**41662** Low-Sociable **2**_AG1 AG **10.66666667**

**41662** Low-Sociable **3**_NS2 NS **12.15189873**

**41662** Low-Sociable **4**_AG2 AG **11.53846154**

**41662** Low-Sociable **5**_NS3 NS **10.4**

**41662** Low-Sociable **6**_AG3 AG **17.74647887**

**41662** Low-Sociable **7**_NS4 NS **15.83333333**

**42039** Low-Sociable **1**_NS1 NS **9.473684211**

**42039** Low-Sociable **2**_AG1 AG **4.666666667**

**42039** Low-Sociable **3**_NS2 NS **9.873417722**

**42039** Low-Sociable **4**_AG2 AG **11.53846154**

**42039** Low-Sociable **5**_NS3 NS **8**

**42039** Low-Sociable **6**_AG3 AG **16.05633803**

**42039** Low-Sociable **7**_NS4 NS **9.166666667**

**42127** High-Sociable **1**_NS1 NS **7.368421053**

**42127** High-Sociable **2**_AG1 AG **19.33333333**

**42127** High-Sociable **3**_NS2 NS **18.2278481**

**42127** High-Sociable **4**_AG2 AG **18.46153846**

**42127** High-Sociable **5**_NS3 NS **17.6**

**42127** High-Sociable **6**_AG3 AG **18.5915493**

**42127** High-Sociable **7**_NS4 NS **21.66666667**

**42156** High-Sociable **1**_NS1 NS **11.57894737**

**42156** High-Sociable **2**_AG1 AG **28.66666667**

**42156** High-Sociable **3**_NS2 NS **31.89873418**

**42156** High-Sociable **4**_AG2 AG **30.76923077**

**42156** High-Sociable **5**_NS3 NS **24**

**42156** High-Sociable **6**_AG3 AG **31.26760563**

**42156** High-Sociable **7**_NS4 NS **35**

**42183** Low-Sociable **1**_NS1 NS **14.73684211**

**42183** Low-Sociable **2**_AG1 AG **19.33333333**

**42183** Low-Sociable **3**_NS2 NS **14.43037975**

**42183** Low-Sociable **4**_AG2 AG **13.84615385**

**42183** Low-Sociable **5**_NS3 NS **11.2**

**42183** Low-Sociable **6**_AG3 AG **5.915492958**

**42183** Low-Sociable **7**_NS4 NS **10.83333333**

**42283** Low-Sociable **1**_NS1 NS **16.84210526**

**42283** Low-Sociable **2**_AG1 AG **13.33333333**

**42283** Low-Sociable **3**_NS2 NS **12.15189873**

**42283** Low-Sociable **4**_AG2 AG **14.61538462**

**42283** Low-Sociable **5**_NS3 NS **9.6**

**42283** Low-Sociable **6**_AG3 AG **21.12676056**

**42283** Low-Sociable **7**_NS4 NS **14.16666667**

**42299** Low-Sociable **1**_NS1 NS **11.57894737**

**42299** Low-Sociable **2**_AG1 AG **18**

**42299** Low-Sociable **3**_NS2 NS **13.67088608**

**42299** Low-Sociable **4**_AG2 AG **20.38461538**

**42299** Low-Sociable **5**_NS3 NS **12.8**

**42299** Low-Sociable **6**_AG3 AG **22.81690141**

**42299** Low-Sociable **7**_NS4 NS **17.5**

**42324** High-Sociable **1**_NS1 NS **13.68421053**

**42324** High-Sociable **2**_AG1 AG **14**

**42324** High-Sociable **3**_NS2 NS **11.39240506**

**42324** High-Sociable **4**_AG2 AG **12.30769231**

**42324** High-Sociable **5**_NS3 NS **11.2**

**42324** High-Sociable **6**_AG3 AG **12.67605634**

**42324** High-Sociable **7**_NS4 NS **10.83333333**

**42333** High-Sociable **1**_NS1 NS **16.84210526**

**42333** High-Sociable **2**_AG1 AG **18**

**42333** High-Sociable **3**_NS2 NS **23.5443038**

**42333** High-Sociable **4**_AG2 AG **28.07692308**

**42333** High-Sociable **5**_NS3 NS **26.4**

**42333** High-Sociable **6**_AG3 AG **25.35211268**

**42333** High-Sociable **7**_NS4 NS **25.83333333**

**42369** High-Sociable **1**_NS1 NS **20**

**42369** High-Sociable **2**_AG1 AG **23.33333333**

**42369** High-Sociable **3**_NS2 NS **23.5443038**

**42369** High-Sociable **4**_AG2 AG **27.30769231**

**42369** High-Sociable **5**_NS3 NS **35.2**

**42369** High-Sociable **6**_AG3 AG **29.57746479**

**42369** High-Sociable **7**_NS4 NS **27.5**

**42371** Low-Sociable **1**_NS1 NS **12.63157895**

**42371** Low-Sociable **2**_AG1 AG **10**

**42371** Low-Sociable **3**_NS2 NS **13.67088608**

**42371** Low-Sociable **4**_AG2 AG **6.538461538**

**42371** Low-Sociable **5**_NS3 NS **12.8**

**42371** Low-Sociable **6**_AG3 AG **9.295774648**

**42371** Low-Sociable **7**_NS4 NS **12.5**

**42373** Low-Sociable **1**_NS1 NS **11.57894737**

**42373** Low-Sociable **2**_AG1 AG **12.66666667**

**42373** Low-Sociable **3**_NS2 NS **6.075949367**

**42373** Low-Sociable **4**_AG2 AG **8.846153846**

**42373** Low-Sociable **5**_NS3 NS **2.4**

**42373** Low-Sociable **6**_AG3 AG **10.14084507**

**42373** Low-Sociable **7**_NS4 NS **4.166666667**

**42396** Low-Sociable **1**_NS1 NS **13.68421053**

**42396** Low-Sociable **2**_AG1 AG **8.666666667**

**42396** Low-Sociable **3**_NS2 NS **12.91139241**

**42396** Low-Sociable **4**_AG2 AG **19.23076923**

**42396** Low-Sociable **5**_NS3 NS **17.6**

**42396** Low-Sociable **6**_AG3 AG **16.90140845**

**42396** Low-Sociable **7**_NS4 NS **20**

**42399** High-Sociable **1**_NS1 NS **13.68421053**

**42399** High-Sociable **2**_AG1 AG **11.33333333**

**42399** High-Sociable **3**_NS2 NS **9.113924051**

**42399** High-Sociable **4**_AG2 AG **23.07692308**

**42399** High-Sociable **5**_NS3 NS **15.2**

**42399** High-Sociable **6**_AG3 AG **18.5915493**

**42399** High-Sociable **7**_NS4 NS **17.5**

**42402** Low-Sociable **1**_NS1 NS **7.368421053**

**42402** Low-Sociable **2**_AG1 AG **13.33333333**

**42402** Low-Sociable **3**_NS2 NS **19.74683544**

**42402** Low-Sociable **4**_AG2 AG **18.84615385**

**42402** Low-Sociable **5**_NS3 NS **22.4**

**42402** Low-Sociable **6**_AG3 AG **17.74647887**

**42402** Low-Sociable **7**_NS4 NS **24.16666667**

**42612** Low-Sociable **1**_NS1 NS **8.421052632**

**42612** Low-Sociable **2**_AG1 AG **18.66666667**

**42612** Low-Sociable **3**_NS2 NS **22.02531646**

**42612** Low-Sociable **4**_AG2 AG **20.38461538**

**42612** Low-Sociable **5**_NS3 NS **26.4**

**42612** Low-Sociable **6**_AG3 AG **21.12676056**

**42612** Low-Sociable **7**_NS4 NS **20**

**42672** High-Sociable **1**_NS1 NS **12.63157895**

**42672** High-Sociable **2**_AG1 AG **20**

**42672** High-Sociable **3**_NS2 NS **13.67088608**

**42672** High-Sociable **4**_AG2 AG **21.92307692**

**42672** High-Sociable **5**_NS3 NS **15.2**

**42672** High-Sociable **6**_AG3 AG **30.42253521**

**42672** High-Sociable **7**_NS4 NS **15.83333333**

**42694** Low-Sociable **1**_NS1 NS **11.57894737**

**42694** Low-Sociable **2**_AG1 AG **8**

**42694** Low-Sociable **3**_NS2 NS **4.556962025**

**42694** Low-Sociable **4**_AG2 AG **9.615384615**

**42694** Low-Sociable **5**_NS3 NS **8.8**

**42694** Low-Sociable **6**_AG3 AG **11.83098592**

**42694** Low-Sociable **7**_NS4 NS **10**

**42705** Low-Sociable **1**_NS1 NS **11.57894737**

**42705** Low-Sociable **2**_AG1 AG **11.33333333**

**42705** Low-Sociable **3**_NS2 NS **4.556962025**

**42705** Low-Sociable **4**_AG2 AG **1.923076923**

**42705** Low-Sociable **5**_NS3 NS **0.8**

**42705** Low-Sociable **6**_AG3 AG **1.690140845**

**42705** Low-Sociable **7**_NS4 NS **4.166666667**

**42717** Low-Sociable **1**_NS1 NS **15.78947368**

**42717** Low-Sociable **2**_AG1 AG **22**

**42717** Low-Sociable **3**_NS2 NS **23.5443038**

**42717** Low-Sociable **4**_AG2 AG **28.07692308**

**42717** Low-Sociable **5**_NS3 NS **24.8**

**42717** Low-Sociable **6**_AG3 AG **29.57746479**

**42717** Low-Sociable **7**_NS4 NS **27.5**

**42723** High-Sociable **1**_NS1 NS **8.421052632**

**42723** High-Sociable **2**_AG1 AG **16.66666667**

**42723** High-Sociable **3**_NS2 NS **17.46835443**

**42723** High-Sociable **4**_AG2 AG **16.53846154**

**42723** High-Sociable **5**_NS3 NS **14.4**

**42723** High-Sociable **6**_AG3 AG **18.5915493**

**42723** High-Sociable **7**_NS4 NS **20**

**42725** High-Sociable **1**_NS1 NS **11.57894737**

**42725** High-Sociable **2**_AG1 AG **9.333333333**

**42725** High-Sociable **3**_NS2 NS **19.74683544**

**42725** High-Sociable **4**_AG2 AG **23.07692308**

**42725** High-Sociable **5**_NS3 NS **16**

**42725** High-Sociable **6**_AG3 AG **22.81690141**

**42725** High-Sociable **7**_NS4 NS **20.83333333**

**42858** Low-Sociable **1**_NS1 NS **10.52631579**

**42858** Low-Sociable **2**_AG1 AG **14**

**42858** Low-Sociable **3**_NS2 NS **9.113924051**

**42858** Low-Sociable **4**_AG2 AG **8.076923077**

**42858** Low-Sociable **5**_NS3 NS **12**

**42858** Low-Sociable **6**_AG3 AG **12.67605634**

**42858** Low-Sociable **7**_NS4 NS **15**

**42979** High-Sociable **1**_NS1 NS **9.473684211**

**42979** High-Sociable **2**_AG1 AG **16**

**42979** High-Sociable **3**_NS2 NS **4.556962025**

**42979** High-Sociable **4**_AG2 AG **11.92307692**

**42979** High-Sociable **5**_NS3 NS **7.2**

**42979** High-Sociable **6**_AG3 AG **7.605633803**

**42979** High-Sociable **7**_NS4 NS **3.333333333**

**43131** Low-Sociable **1**_NS1 NS **10.52631579**

**43131** Low-Sociable **2**_AG1 AG **12.66666667**

**43131** Low-Sociable **3**_NS2 NS **19.74683544**

**43131** Low-Sociable **4**_AG2 AG **15.76923077**

**43131** Low-Sociable **5**_NS3 NS **18.4**

**43131** Low-Sociable **6**_AG3 AG **19.43661972**

**43131** Low-Sociable **7**_NS4 NS **16.66666667**

**43157** Low-Sociable **1**_NS1 NS **9.473684211**

**43157** Low-Sociable **2**_AG1 AG **7.333333333**

**43157** Low-Sociable **3**_NS2 NS **11.39240506**

**43157** Low-Sociable **4**_AG2 AG **15**

**43157** Low-Sociable **5**_NS3 NS **17.6**

**43157** Low-Sociable **6**_AG3 AG **16.90140845**

**43157** Low-Sociable **7**_NS4 NS **22.5**

**43215** High-Sociable **1**_NS1 NS **11.57894737**

**43215** High-Sociable **2**_AG1 AG **20**

**43215** High-Sociable **3**_NS2 NS **13.67088608**

**43215** High-Sociable **4**_AG2 AG **11.92307692**

**43215** High-Sociable **5**_NS3 NS **11.2**

**43215** High-Sociable **6**_AG3 AG **10.14084507**

**43215** High-Sociable **7**_NS4 NS **7.5**

**43219** Low-Sociable **1**_NS1 NS **14.73684211**

**43219** Low-Sociable **2**_AG1 AG **21.33333333**

**43219** Low-Sociable **3**_NS2 NS **27.34177215**

**43219** Low-Sociable **4**_AG2 AG **22.69230769**

**43219** Low-Sociable **5**_NS3 NS **24**

**43219** Low-Sociable **6**_AG3 AG **21.97183099**

**43219** Low-Sociable **7**_NS4 NS **27.5**

**43221** High-Sociable **1**_NS1 NS **13.68421053**

**43221** High-Sociable **2**_AG1 AG **23.33333333**

**43221** High-Sociable **3**_NS2 NS **28.86075949**

**43221** High-Sociable **4**_AG2 AG **25**

**43221** High-Sociable **5**_NS3 NS **22.4**

**43221** High-Sociable **6**_AG3 AG **26.1971831**

**43221** High-Sociable **7**_NS4 NS **30**

**43292** High-Sociable **1**_NS1 NS **9.473684211**

**43292** High-Sociable **2**_AG1 AG **20**

**43292** High-Sociable **3**_NS2 NS **15.18987342**

**43292** High-Sociable **4**_AG2 AG **24.23076923**

**43292** High-Sociable **5**_NS3 NS **15.2**

**43292** High-Sociable **6**_AG3 AG **20.28169014**

**43292** High-Sociable **7**_NS4 NS **13.33333333**

**43298** High-Sociable **1**_NS1 NS **10.52631579**

**43298** High-Sociable **2**_AG1 AG **12**

**43298** High-Sociable **3**_NS2 NS **15.18987342**

**43298** High-Sociable **4**_AG2 AG **10.38461538**

**43298** High-Sociable **5**_NS3 NS **5.6**

**43298** High-Sociable **6**_AG3 AG **10.98591549**

**43298** High-Sociable **7**_NS4 NS **9.166666667**

**43300** High-Sociable **1**_NS1 NS **6.315789474**

**43300** High-Sociable **2**_AG1 AG **14**

**43300** High-Sociable **3**_NS2 NS **14.43037975**

**43300** High-Sociable **4**_AG2 AG **16.53846154**

**43300** High-Sociable **5**_NS3 NS **16**

**43300** High-Sociable **6**_AG3 AG **16.05633803**

**43300** High-Sociable **7**_NS4 NS **15**

**43395** High-Sociable **1**_NS1 NS **7.368421053**

**43395** High-Sociable **2**_AG1 AG **10**

**43395** High-Sociable **3**_NS2 NS **3.037974684**

**43395** High-Sociable **4**_AG2 AG **3.846153846**

**43395** High-Sociable **5**_NS3 NS **0.8**

**43395** High-Sociable **6**_AG3 AG **12.67605634**

**43395** High-Sociable **7**_NS4 NS **11.66666667**

**43442** Low-Sociable **1**_NS1 NS **17.89473684**

**43442** Low-Sociable **2**_AG1 AG **12**

**43442** Low-Sociable **3**_NS2 NS **6.835443038**

**43442** Low-Sociable **4**_AG2 AG **10**

**43442** Low-Sociable **5**_NS3 NS **8**

**43442** Low-Sociable **6**_AG3 AG **14.36619718**

**43442** Low-Sociable **7**_NS4 NS **11.66666667**

**43449** High-Sociable **1**_NS1 NS **7.368421053**

**43449** High-Sociable **2**_AG1 AG **8.666666667**

**43449** High-Sociable **3**_NS2 NS **1.518987342**

**43449** High-Sociable **4**_AG2 AG **3.846153846**

**43449** High-Sociable **5**_NS3 NS **0.8**

**43449** High-Sociable **6**_AG3 AG **13.52112676**

**43449** High-Sociable **7**_NS4 NS **10.83333333**

**43523** High-Sociable **1**_NS1 NS **11.57894737**

**43523** High-Sociable **2**_AG1 AG **21.33333333**

**43523** High-Sociable **3**_NS2 NS **17.46835443**

**43523** High-Sociable **4**_AG2 AG **23.46153846**

**43523** High-Sociable **5**_NS3 NS **20**

**43523** High-Sociable **6**_AG3 AG **21.97183099**

**43523** High-Sociable **7**_NS4 NS **21.66666667**

**43580** Low-Sociable **1**_NS1 NS **11.57894737**

**43580** Low-Sociable **2**_AG1 AG **12**

**43580** Low-Sociable **3**_NS2 NS **12.91139241**

**43580** Low-Sociable **4**_AG2 AG **14.61538462**

**43580** Low-Sociable **5**_NS3 NS **13.6**

**43580** Low-Sociable **6**_AG3 AG **15.21126761**

**43580** Low-Sociable **7**_NS4 NS **15**

**43581** Low-Sociable **1**_NS1 NS **10.52631579**

**43581** Low-Sociable **2**_AG1 AG **18.66666667**

**43581** Low-Sociable **3**_NS2 NS **15.94936709**

**43581** Low-Sociable **4**_AG2 AG **15.76923077**

**43581** Low-Sociable **5**_NS3 NS **19.2**

**43581** Low-Sociable **6**_AG3 AG **16.90140845**

**43581** Low-Sociable **7**_NS4 NS **20.83333333**

**43637** High-Sociable **1**_NS1 NS **6.315789474**

**43637** High-Sociable **2**_AG1 AG **11.33333333**

**43637** High-Sociable **3**_NS2 NS **9.113924051**

**43637** High-Sociable **4**_AG2 AG **16.53846154**

**43637** High-Sociable **5**_NS3 NS **12**

**43637** High-Sociable **6**_AG3 AG **13.52112676**

**43637** High-Sociable **7**_NS4 NS **17.5**

**43675** High-Sociable **1**_NS1 NS **7.368421053**

**43675** High-Sociable **2**_AG1 AG **16.66666667**

**43675** High-Sociable **3**_NS2 NS **18.2278481**

**43675** High-Sociable **4**_AG2 AG **20.76923077**

**43675** High-Sociable **5**_NS3 NS **17.6**

**43675** High-Sociable **6**_AG3 AG **18.5915493**

**43675** High-Sociable **7**_NS4 NS **17.5**

**43746** High-Sociable **1**_NS1 NS **11.57894737**

**43746** High-Sociable **2**_AG1 AG **20.66666667**

**43746** High-Sociable **3**_NS2 NS **14.43037975**

**43746** High-Sociable **4**_AG2 AG **22.30769231**

**43746** High-Sociable **5**_NS3 NS **14.4**

**43746** High-Sociable **6**_AG3 AG **25.35211268**

**43746** High-Sociable **7**_NS4 NS **19.16666667**

**43854** High-Sociable **1**_NS1 NS **9.473684211**

**43854** High-Sociable **2**_AG1 AG **12.66666667**

**43854** High-Sociable **3**_NS2 NS **12.91139241**

**43854** High-Sociable **4**_AG2 AG **20.76923077**

**43854** High-Sociable **5**_NS3 NS **24**

**43854** High-Sociable **6**_AG3 AG **27.04225352**

**43854** High-Sociable **7**_NS4 NS **19.16666667**

;

**RUN**;

**PROC** **MIXED** ASYCOV NOBOUND DATA=VP_task_repeated_measures_forma ALPHA=**0.05**;

CLASS ID SociabilityText OrderedSection SectionType;

MODEL Look_RPM = SociabilityText OrderedSection(SectionType ) SectionType OrderedSection*SociabilityText(SectionType ) SectionType*SociabilityText/ SOLUTION DDFM=KENWARDROGER;

RANDOM ID(SociabilityText ) ID*SectionType(SociabilityText ) / SOLUTION ;

lsmeans SectionType*SociabilityText / slice=sectiontype slice=sociabilitytext ;

**RUN**;

## Proportion of time spent looking at each exemplar type

**DATA** VP_task_repeated_measures_forma; INPUT ID SociabilityText &$16. OrderedSection &$16. SectionType &$16. Look_Dur2; Lines;

**40737** Low-Sociable **1**_NS1 NS **0.421052632**

**40737** Low-Sociable **2**_AG1 AG **0.411111111**

**40737** Low-Sociable **3**_NS2 NS **0.253164557**

**40737** Low-Sociable **4**_AG2 AG **0.333333333**

**40737** Low-Sociable **5**_NS3 NS **0.2**

**40737** Low-Sociable **6**_AG3 AG **0.126760563**

**40737** Low-Sociable **7**_NS4 NS **0.111111111**

**41470** Low-Sociable **1**_NS1 NS **0.754385965**

**41470** Low-Sociable **2**_AG1 AG **0.844444444**

**41470** Low-Sociable **3**_NS2 NS **0.607594937**

**41470** Low-Sociable **4**_AG2 AG **0.782051282**

**41470** Low-Sociable **5**_NS3 NS **0.64**

**41470** Low-Sociable **6**_AG3 AG **0.845070423**

**41470** Low-Sociable **7**_NS4 NS **0.638888889**

**41475** High-Sociable **1**_NS1 NS **0.666666667**

**41475** High-Sociable **2**_AG1 AG **0.466666667**

**41475** High-Sociable **3**_NS2 NS **0.329113924**

**41475** High-Sociable **4**_AG2 AG **0.679487179**

**41475** High-Sociable **5**_NS3 NS **0.613333333**

**41475** High-Sociable **6**_AG3 AG **0.873239437**

**41475** High-Sociable **7**_NS4 NS **0.708333333**

**41518** High-Sociable **1**_NS1 NS **0.789473684**

**41518** High-Sociable **2**_AG1 AG **0.844444444**

**41518** High-Sociable **3**_NS2 NS **0.569620253**

**41518** High-Sociable **4**_AG2 AG **0.782051282**

**41518** High-Sociable **5**_NS3 NS **0.733333333**

**41518** High-Sociable **6**_AG3 AG **0.873239437**

**41518** High-Sociable **7**_NS4 NS **0.694444444**

**41520** Low-Sociable **1**_NS1 NS **0.50877193**

**41520** Low-Sociable **2**_AG1 AG **0.722222222**

**41520** Low-Sociable **3**_NS2 NS **0.430379747**

**41520** Low-Sociable **4**_AG2 AG **0.878205128**

**41520** Low-Sociable **5**_NS3 NS **0.506666667**

**41520** Low-Sociable **6**_AG3 AG **0.943661972**

**41520** Low-Sociable **7**_NS4 NS **0.611111111**

**41566** High-Sociable **1**_NS1 NS **0.596491228**

**41566** High-Sociable **2**_AG1 AG **0.566666667**

**41566** High-Sociable **3**_NS2 NS **0.392405063**

**41566** High-Sociable **4**_AG2 AG **0.807692308**

**41566** High-Sociable **5**_NS3 NS **0.893333333**

**41566** High-Sociable **6**_AG3 AG **0.887323944**

**41566** High-Sociable **7**_NS4 NS **0.625**

**41569** Low-Sociable **1**_NS1 NS **0.421052632**

**41569** Low-Sociable **2**_AG1 AG **0.5**

**41569** Low-Sociable **3**_NS2 NS **0.265822785**

**41569** Low-Sociable **4**_AG2 AG **0.769230769**

**41569** Low-Sociable **5**_NS3 NS **0.306666667**

**41569** Low-Sociable **6**_AG3 AG **0.85915493**

**41569** Low-Sociable **7**_NS4 NS **0.347222222**

**41659** Low-Sociable **1**_NS1 NS **0.649122807**

**41659** Low-Sociable **2**_AG1 AG **0.722222222**

**41659** Low-Sociable **3**_NS2 NS **0.683544304**

**41659** Low-Sociable **4**_AG2 AG **0.852564103**

**41659** Low-Sociable **5**_NS3 NS **0.453333333**

**41659** Low-Sociable **6**_AG3 AG **0.873239437**

**41659** Low-Sociable **7**_NS4 NS **0.527777778**

**41662** Low-Sociable **1**_NS1 NS **0.614035088**

**41662** Low-Sociable **2**_AG1 AG **0.722222222**

**41662** Low-Sociable **3**_NS2 NS **0.708860759**

**41662** Low-Sociable **4**_AG2 AG **0.858974359**

**41662** Low-Sociable **5**_NS3 NS **0.68**

**41662** Low-Sociable **6**_AG3 AG **0.732394366**

**41662** Low-Sociable **7**_NS4 NS **0.541666667**

**42039** Low-Sociable **1**_NS1 NS **0.368421053**

**42039** Low-Sociable **2**_AG1 AG **0.655555556**

**42039** Low-Sociable **3**_NS2 NS **0.189873418**

**42039** Low-Sociable **4**_AG2 AG **0.66025641**

**42039** Low-Sociable **5**_NS3 NS **0.186666667**

**42039** Low-Sociable **6**_AG3 AG **0.676056338**

**42039** Low-Sociable **7**_NS4 NS **0.305555556**

**42127** High-Sociable **1**_NS1 NS **0.877192982**

**42127** High-Sociable **2**_AG1 AG **0.733333333**

**42127** High-Sociable **3**_NS2 NS **0.518987342**

**42127** High-Sociable **4**_AG2 AG **0.717948718**

**42127** High-Sociable **5**_NS3 NS **0.666666667**

**42127** High-Sociable **6**_AG3 AG **0.830985915**

**42127** High-Sociable **7**_NS4 NS **0.666666667**

**42156** High-Sociable **1**_NS1 NS **0.754385965**

**42156** High-Sociable **2**_AG1 AG **0.744444444**

**42156** High-Sociable **3**_NS2 NS **0.607594937**

**42156** High-Sociable **4**_AG2 AG **0.717948718**

**42156** High-Sociable **5**_NS3 NS **0.56**

**42156** High-Sociable **6**_AG3 AG **0.732394366**

**42156** High-Sociable **7**_NS4 NS **0.597222222**

**42183** Low-Sociable **1**_NS1 NS **0.701754386**

**42183** Low-Sociable **2**_AG1 AG **0.6**

**42183** Low-Sociable **3**_NS2 NS **0.405063291**

**42183** Low-Sociable **4**_AG2 AG **0.326923077**

**42183** Low-Sociable **5**_NS3 NS **0.306666667**

**42183** Low-Sociable **6**_AG3 AG **0.14084507**

**42183** Low-Sociable **7**_NS4 NS **0.208333333**

**42283** Low-Sociable **1**_NS1 NS **0.421052632**

**42283** Low-Sociable **2**_AG1 AG **0.466666667**

**42283** Low-Sociable **3**_NS2 NS **0.291139241**

**42283** Low-Sociable **4**_AG2 AG **0.698717949**

**42283** Low-Sociable **5**_NS3 NS **0.413333333**

**42283** Low-Sociable **6**_AG3 AG **0.690140845**

**42283** Low-Sociable **7**_NS4 NS **0.5**

**42299** Low-Sociable **1**_NS1 NS **0.754385965**

**42299** Low-Sociable **2**_AG1 AG **0.633333333**

**42299** Low-Sociable **3**_NS2 NS **0.46835443**

**42299** Low-Sociable **4**_AG2 AG **0.467948718**

**42299** Low-Sociable **5**_NS3 NS **0.306666667**

**42299** Low-Sociable **6**_AG3 AG **0.563380282**

**42299** Low-Sociable **7**_NS4 NS **0.388888889**

**42324** High-Sociable **1**_NS1 NS **0.631578947**

**42324** High-Sociable **2**_AG1 AG **0.611111111**

**42324** High-Sociable **3**_NS2 NS **0.405063291**

**42324** High-Sociable **4**_AG2 AG **0.634615385**

**42324** High-Sociable **5**_NS3 NS **0.546666667**

**42324** High-Sociable **6**_AG3 AG **0.436619718**

**42324** High-Sociable **7**_NS4 NS **0.375**

**42333** High-Sociable **1**_NS1 NS **0.631578947**

**42333** High-Sociable **2**_AG1 AG **0.811111111**

**42333** High-Sociable **3**_NS2 NS **0.632911392**

**42333** High-Sociable **4**_AG2 AG **0.679487179**

**42333** High-Sociable **5**_NS3 NS **0.64**

**42333** High-Sociable **6**_AG3 AG **0.718309859**

**42333** High-Sociable **7**_NS4 NS **0.694444444**

**42369** High-Sociable **1**_NS1 NS **0.754385965**

**42369** High-Sociable **2**_AG1 AG **0.822222222**

**42369** High-Sociable **3**_NS2 NS **0.607594937**

**42369** High-Sociable **4**_AG2 AG **0.801282051**

**42369** High-Sociable **5**_NS3 NS **0.52**

**42369** High-Sociable **6**_AG3 AG **0.830985915**

**42369** High-Sociable **7**_NS4 NS **0.513888889**

**42371** Low-Sociable **1**_NS1 NS **0.543859649**

**42371** Low-Sociable **2**_AG1 AG **0.8**

**42371** Low-Sociable **3**_NS2 NS **0.582278481**

**42371** Low-Sociable **4**_AG2 AG **0.929487179**

**42371** Low-Sociable **5**_NS3 NS **0.64**

**42371** Low-Sociable **6**_AG3 AG **0.887323944**

**42371** Low-Sociable **7**_NS4 NS **0.541666667**

**42373** Low-Sociable **1**_NS1 NS **0.561403509**

**42373** Low-Sociable **2**_AG1 AG **0.455555556**

**42373** Low-Sociable **3**_NS2 NS **0.253164557**

**42373** Low-Sociable **4**_AG2 AG **0.435897436**

**42373** Low-Sociable **5**_NS3 NS **0.026666667**

**42373** Low-Sociable **6**_AG3 AG **0.197183099**

**42373** Low-Sociable **7**_NS4 NS **0.083333333**

**42396** Low-Sociable **1**_NS1 NS **0.789473684**

**42396** Low-Sociable **2**_AG1 AG **0.377777778**

**42396** Low-Sociable **3**_NS2 NS **0.392405063**

**42396** Low-Sociable **4**_AG2 AG **0.634615385**

**42396** Low-Sociable **5**_NS3 NS **0.373333333**

**42396** Low-Sociable **6**_AG3 AG **0.718309859**

**42396** Low-Sociable **7**_NS4 NS **0.638888889**

**42399** High-Sociable **1**_NS1 NS **0.666666667**

**42399** High-Sociable **2**_AG1 AG **0.644444444**

**42399** High-Sociable **3**_NS2 NS **0.53164557**

**42399** High-Sociable **4**_AG2 AG **0.576923077**

**42399** High-Sociable **5**_NS3 NS **0.626666667**

**42399** High-Sociable **6**_AG3 AG **0.746478873**

**42399** High-Sociable **7**_NS4 NS **0.5**

**42402** Low-Sociable **1**_NS1 NS **0.368421053**

**42402** Low-Sociable **2**_AG1 AG **0.822222222**

**42402** Low-Sociable **3**_NS2 NS **0.835443038**

**42402** Low-Sociable **4**_AG2 AG **0.903846154**

**42402** Low-Sociable **5**_NS3 NS **0.666666667**

**42402** Low-Sociable **6**_AG3 AG **0.901408451**

**42402** Low-Sociable **7**_NS4 NS **0.819444444**

**42612** Low-Sociable **1**_NS1 NS **0.385964912**

**42612** Low-Sociable **2**_AG1 AG **0.7**

**42612** Low-Sociable **3**_NS2 NS **0.759493671**

**42612** Low-Sociable **4**_AG2 AG **0.935897436**

**42612** Low-Sociable **5**_NS3 NS **0.76**

**42612** Low-Sociable **6**_AG3 AG **0.929577465**

**42612** Low-Sociable **7**_NS4 NS **0.763888889**

**42672** High-Sociable **1**_NS1 NS **0.649122807**

**42672** High-Sociable **2**_AG1 AG **0.655555556**

**42672** High-Sociable **3**_NS2 NS **0.569620253**

**42672** High-Sociable **4**_AG2 AG **0.685897436**

**42672** High-Sociable **5**_NS3 NS **0.306666667**

**42672** High-Sociable **6**_AG3 AG **0.478873239**

**42672** High-Sociable **7**_NS4 NS **0.402777778**

**42694** Low-Sociable **1**_NS1 NS **0.350877193**

**42694** Low-Sociable **2**_AG1 AG **0.355555556**

**42694** Low-Sociable **3**_NS2 NS **0.215189873**

**42694** Low-Sociable **4**_AG2 AG **0.192307692**

**42694** Low-Sociable **5**_NS3 NS **0.146666667**

**42694** Low-Sociable **6**_AG3 AG **0.464788732**

**42694** Low-Sociable **7**_NS4 NS **0.180555556**

**42705** Low-Sociable **1**_NS1 NS **0.245614035**

**42705** Low-Sociable **2**_AG1 AG **0.222222222**

**42705** Low-Sociable **3**_NS2 NS **0.075949367**

**42705** Low-Sociable **4**_AG2 AG **0.038461538**

**42705** Low-Sociable **5**_NS3 NS **0**

**42705** Low-Sociable **6**_AG3 AG **0.014084507**

**42705** Low-Sociable **7**_NS4 NS **0.055555556**

**42717** Low-Sociable **1**_NS1 NS **0.649122807**

**42717** Low-Sociable **2**_AG1 AG **0.711111111**

**42717** Low-Sociable **3**_NS2 NS **0.683544304**

**42717** Low-Sociable **4**_AG2 AG **0.621794872**

**42717** Low-Sociable **5**_NS3 NS **0.573333333**

**42717** Low-Sociable **6**_AG3 AG **0.661971831**

**42717** Low-Sociable **7**_NS4 NS **0.5**

**42723** High-Sociable **1**_NS1 NS **0.771929825**

**42723** High-Sociable **2**_AG1 AG **0.855555556**

**42723** High-Sociable **3**_NS2 NS **0.658227848**

**42723** High-Sociable **4**_AG2 AG **0.826923077**

**42723** High-Sociable **5**_NS3 NS **0.573333333**

**42723** High-Sociable **6**_AG3 AG **0.802816901**

**42723** High-Sociable **7**_NS4 NS **0.680555556**

**42725** High-Sociable **1**_NS1 NS **0.333333333**

**42725** High-Sociable **2**_AG1 AG **0.455555556**

**42725** High-Sociable **3**_NS2 NS **0.417721519**

**42725** High-Sociable **4**_AG2 AG **0.737179487**

**42725** High-Sociable **5**_NS3 NS **0.48**

**42725** High-Sociable **6**_AG3 AG **0.661971831**

**42725** High-Sociable **7**_NS4 NS **0.5**

**42858** Low-Sociable **1**_NS1 NS **0.50877193**

**42858** Low-Sociable **2**_AG1 AG **0.6**

**42858** Low-Sociable **3**_NS2 NS **0.253164557**

**42858** Low-Sociable **4**_AG2 AG **0.730769231**

**42858** Low-Sociable **5**_NS3 NS **0.506666667**

**42858** Low-Sociable **6**_AG3 AG **0.774647887**

**42858** Low-Sociable **7**_NS4 NS **0.416666667**

**42979** High-Sociable **1**_NS1 NS **0.368421053**

**42979** High-Sociable **2**_AG1 AG **0.333333333**

**42979** High-Sociable **3**_NS2 NS **0.215189873**

**42979** High-Sociable **4**_AG2 AG **0.358974359**

**42979** High-Sociable **5**_NS3 NS **0.146666667**

**42979** High-Sociable **6**_AG3 AG **0.126760563**

**42979** High-Sociable **7**_NS4 NS **0.055555556**

**43131** Low-Sociable **1**_NS1 NS **0.929824561**

**43131** Low-Sociable **2**_AG1 AG **0.9**

**43131** Low-Sociable **3**_NS2 NS **0.683544304**

**43131** Low-Sociable **4**_AG2 AG **0.814102564**

**43131** Low-Sociable **5**_NS3 NS **0.68**

**43131** Low-Sociable **6**_AG3 AG **0.802816901**

**43131** Low-Sociable **7**_NS4 NS **0.611111111**

**43157** Low-Sociable **1**_NS1 NS **0.333333333**

**43157** Low-Sociable **2**_AG1 AG **0.477777778**

**43157** Low-Sociable **3**_NS2 NS **0.379746835**

**43157** Low-Sociable **4**_AG2 AG **0.801282051**

**43157** Low-Sociable **5**_NS3 NS **0.52**

**43157** Low-Sociable **6**_AG3 AG **0.774647887**

**43157** Low-Sociable **7**_NS4 NS **0.569444444**

**43215** High-Sociable **1**_NS1 NS **0.614035088**

**43215** High-Sociable **2**_AG1 AG **0.5**

**43215** High-Sociable **3**_NS2 NS **0.227848101**

**43215** High-Sociable **4**_AG2 AG **0.230769231**

**43215** High-Sociable **5**_NS3 NS **0.146666667**

**43215** High-Sociable **6**_AG3 AG **0.126760563**

**43215** High-Sociable **7**_NS4 NS **0.097222222**

**43219** Low-Sociable **1**_NS1 NS **0.614035088**

**43219** Low-Sociable **2**_AG1 AG **0.788888889**

**43219** Low-Sociable **3**_NS2 NS **0.620253165**

**43219** Low-Sociable **4**_AG2 AG **0.75**

**43219** Low-Sociable **5**_NS3 NS **0.613333333**

**43219** Low-Sociable **6**_AG3 AG **0.774647887**

**43219** Low-Sociable **7**_NS4 NS **0.611111111**

**43221** High-Sociable **1**_NS1 NS **0.842105263**

**43221** High-Sociable **2**_AG1 AG **0.811111111**

**43221** High-Sociable **3**_NS2 NS **0.696202532**

**43221** High-Sociable **4**_AG2 AG **0.756410256**

**43221** High-Sociable **5**_NS3 NS **0.653333333**

**43221** High-Sociable **6**_AG3 AG **0.788732394**

**43221** High-Sociable **7**_NS4 NS **0.666666667**

**43292** High-Sociable **1**_NS1 NS **0.894736842**

**43292** High-Sociable **2**_AG1 AG **0.766666667**

**43292** High-Sociable **3**_NS2 NS **0.886075949**

**43292** High-Sociable **4**_AG2 AG **0.782051282**

**43292** High-Sociable **5**_NS3 NS **0.8**

**43292** High-Sociable **6**_AG3 AG **0.816901408**

**43292** High-Sociable **7**_NS4 NS **0.805555556**

**43298** High-Sociable **1**_NS1 NS **0.49122807**

**43298** High-Sociable **2**_AG1 AG **0.544444444**

**43298** High-Sociable **3**_NS2 NS **0.556962025**

**43298** High-Sociable **4**_AG2 AG **0.461538462**

**43298** High-Sociable **5**_NS3 NS **0.133333333**

**43298** High-Sociable **6**_AG3 AG **0.507042254**

**43298** High-Sociable **7**_NS4 NS **0.319444444**

**43300** High-Sociable **1**_NS1 NS **0.666666667**

**43300** High-Sociable **2**_AG1 AG **0.766666667**

**43300** High-Sociable **3**_NS2 NS **0.569620253**

**43300** High-Sociable **4**_AG2 AG **0.641025641**

**43300** High-Sociable **5**_NS3 NS **0.6**

**43300** High-Sociable **6**_AG3 AG **0.704225352**

**43300** High-Sociable **7**_NS4 NS **0.638888889**

**43395** High-Sociable **1**_NS1 NS **0.333333333**

**43395** High-Sociable **2**_AG1 AG **0.288888889**

**43395** High-Sociable **3**_NS2 NS **0.025316456**

**43395** High-Sociable **4**_AG2 AG **0.057692308**

**43395** High-Sociable **5**_NS3 NS **0**

**43395** High-Sociable **6**_AG3 AG **0.394366197**

**43395** High-Sociable **7**_NS4 NS **0.222222222**

**43442** Low-Sociable **1**_NS1 NS **0.473684211**

**43442** Low-Sociable **2**_AG1 AG **0.266666667**

**43442** Low-Sociable **3**_NS2 NS **0.126582278**

**43442** Low-Sociable **4**_AG2 AG **0.224358974**

**43442** Low-Sociable **5**_NS3 NS **0.146666667**

**43442** Low-Sociable **6**_AG3 AG **0.352112676**

**43442** Low-Sociable **7**_NS4 NS **0.277777778**

**43449** High-Sociable **1**_NS1 NS **0.315789474**

**43449** High-Sociable **2**_AG1 AG **0.266666667**

**43449** High-Sociable **3**_NS2 NS **0.012658228**

**43449** High-Sociable **4**_AG2 AG **0.076923077**

**43449** High-Sociable **5**_NS3 NS **0**

**43449** High-Sociable **6**_AG3 AG **0.408450704**

**43449** High-Sociable **7**_NS4 NS **0.236111111**

**43523** High-Sociable **1**_NS1 NS **0.526315789**

**43523** High-Sociable **2**_AG1 AG **0.644444444**

**43523** High-Sociable **3**_NS2 NS **0.53164557**

**43523** High-Sociable **4**_AG2 AG **0.673076923**

**43523** High-Sociable **5**_NS3 NS **0.373333333**

**43523** High-Sociable **6**_AG3 AG **0.718309859**

**43523** High-Sociable **7**_NS4 NS **0.583333333**

**43580** Low-Sociable **1**_NS1 NS **0.631578947**

**43580** Low-Sociable **2**_AG1 AG **0.566666667**

**43580** Low-Sociable **3**_NS2 NS **0.291139241**

**43580** Low-Sociable **4**_AG2 AG **0.596153846**

**43580** Low-Sociable **5**_NS3 NS **0.373333333**

**43580** Low-Sociable **6**_AG3 AG **0.704225352**

**43580** Low-Sociable **7**_NS4 NS **0.555555556**

**43581** Low-Sociable **1**_NS1 NS **0.631578947**

**43581** Low-Sociable **2**_AG1 AG **0.744444444**

**43581** Low-Sociable **3**_NS2 NS **0.708860759**

**43581** Low-Sociable **4**_AG2 AG **0.762820513**

**43581** Low-Sociable **5**_NS3 NS **0.586666667**

**43581** Low-Sociable **6**_AG3 AG **0.591549296**

**43581** Low-Sociable **7**_NS4 NS **0.611111111**

**43637** High-Sociable **1**_NS1 NS **0.350877193**

**43637** High-Sociable **2**_AG1 AG **0.488888889**

**43637** High-Sociable **3**_NS2 NS **0.367088608**

**43637** High-Sociable **4**_AG2 AG **0.705128205**

**43637** High-Sociable **5**_NS3 NS **0.586666667**

**43637** High-Sociable **6**_AG3 AG **0.746478873**

**43637** High-Sociable **7**_NS4 NS **0.513888889**

**43675** High-Sociable **1**_NS1 NS **0.807017544**

**43675** High-Sociable **2**_AG1 AG **0.722222222**

**43675** High-Sociable **3**_NS2 NS **0.708860759**

**43675** High-Sociable **4**_AG2 AG **0.762820513**

**43675** High-Sociable **5**_NS3 NS **0.573333333**

**43675** High-Sociable **6**_AG3 AG **0.816901408**

**43675** High-Sociable **7**_NS4 NS **0.597222222**

**43746** High-Sociable **1**_NS1 NS **0.736842105**

**43746** High-Sociable **2**_AG1 AG **0.544444444**

**43746** High-Sociable **3**_NS2 NS **0.35443038**

**43746** High-Sociable **4**_AG2 AG **0.557692308**

**43746** High-Sociable **5**_NS3 NS **0.426666667**

**43746** High-Sociable **6**_AG3 AG **0.690140845**

**43746** High-Sociable **7**_NS4 NS **0.472222222**

**43854** High-Sociable **1**_NS1 NS **0.543859649**

**43854** High-Sociable **2**_AG1 AG **0.644444444**

**43854** High-Sociable **3**_NS2 NS **0.417721519**

**43854** High-Sociable **4**_AG2 AG **0.679487179**

**43854** High-Sociable **5**_NS3 NS **0.413333333**

**43854** High-Sociable **6**_AG3 AG **0.633802817**

**43854** High-Sociable **7**_NS4 NS **0.458333333**

;

**RUN**;

**PROC** **MIXED** ASYCOV NOBOUND DATA=VP_task_repeated_measures_forma ALPHA=**0.05**;

CLASS ID SociabilityText OrderedSection SectionType;

MODEL Look_Dur2 = SociabilityText OrderedSection(SectionType ) SectionType OrderedSection*SociabilityText(SectionType ) SectionType*SociabilityText/ SOLUTION DDFM=KENWARDROGER;

RANDOM ID(SociabilityText ) ID*SectionType(SociabilityText ) / SOLUTION ;

lsmeans SectionType*SociabilityText;

**RUN**;

# Multivariate and univariate logistic regression models

**DATA** combined_data_across_tasks_2016; INPUT SociabilityText &$16. _NovelPreferred Gaze_AG_NS Look_AG_NS; Lines;

High-Sociable **0.60847861** **1.04033973765641** **1.243440010206**

High-Sociable **0.60274934** **1.01971544413241** **1.24548013527668**

High-Sociable **0.5300341** **1.0106157410579** **1.40689668704357**

High-Sociable **0.51180942** **1.03919820995911** **1.20451065150894**

High-Sociable **0.59623677** **1.03046884983509** **1.11305062097937**

High-Sociable **0.62001995** **1.05287597170688** **1.31405287812761**

High-Sociable **0.53148939** **1.01843230726694** **1.17224812049245**

High-Sociable **0.54537441** **0.985611609955117** **1.15633340494124**

High-Sociable **0.58583555** **1.05160052315064** **1.20885986437559**

High-Sociable **0.55570029** **1.09473885566574** **1.10091313052784**

High-Sociable **0.57611433** **1.02499634036102** **1.22672808421648**

High-Sociable **0.4923544** **0.999769837322854** **1.21785196861019**

High-Sociable **0.57027522** **1.08008155533614** **1.31505419710021**

High-Sociable **0.57919718** **1.03828796107955** **1.27692436292189**

High-Sociable **0.57639554** **1.03374064369018** **1.16235701530929**

High-Sociable **0.60458685** **1.08697974075653** **1.13150311716633**

High-Sociable **0.59050141** **1.03204808190296** **1.37127129016458**

High-Sociable **0.55914866** **1.04907120666734** **1.25067302619411**

High-Sociable **0.57523409** **1.09806070862419** **1.47402329493161**

High-Sociable **0.59376951** **1.09405653276161** **1.48526953662217**

High-Sociable **0.52532357** **1.04631159355463** **1.15672223262969**

High-Sociable **0.52833874** **1.05913474060248** **1.2854668106603**

High-Sociable **0.56005251** **1.03234293583182** **1.19881814092794**

High-Sociable **0.53313672** **1.06006469876822** **1.12578608808519**

High-Sociable **0.51169277** **1.04879546138961** **1.18100299321039**

Low-Sociable **0.49308231** **0.971057351026418** **0.977792276938021**

Low-Sociable **0.55544451** **0.920904274546403** **1.05829764064303**

Low-Sociable **0.51046897** **0.913421679752892** **1.04714308805682**

Low-Sociable **0.4793424** **0.935253477350035** **1.01760344325476**

Low-Sociable **0.42884513** **0.916968136260035** **1.0883318439687**

Low-Sociable **0.46534864** **0.934780385585545** **1.0016471418493**

Low-Sociable **0.50059243** **0.947098018023182** **0.984683633783884**

Low-Sociable **0.42468119** **0.960251019042284** **1.01106118299268**

Low-Sociable **0.4293038** **0.967673074578569** **0.969477580139553**

Low-Sociable **0.50078912** **1.01196389437594** **0.933832338863412**

Low-Sociable **0.56283585** **0.901559743574389** **1.09746349326584**

Low-Sociable **0.49723934** **1.0128721804593** **0.914607106675521**

Low-Sociable **0.45035681** **0.958531533249498** **1.0282811322969**

Low-Sociable **0.48427298** **0.902919830763969** **1.03373320031328**

Low-Sociable **0.53334431** **0.885518894977245** **1.00262534049676**

Low-Sociable **0.52029297** **0.974892429749172** **0.994920100138041**

Low-Sociable **0.39872045** **0.916157632388886** **1.03659686108868**

Low-Sociable **0.49286846** **0.988112746455762** **0.979151890186553**

Low-Sociable **0.49781463** **0.936944199742065** **1.01699416110856**

Low-Sociable **0.49476141** **0.94990642626122** **1.01786609183636**

Low-Sociable **0.48366138** **0.930332924383194** **1.04561170927462**

Low-Sociable **0.51572254** **0.942716376319601** **1.02399955969656**

Low-Sociable **0.49085726** **0.960584215476345** **0.997845159867441**

Low-Sociable **0.42713188** **0.962689225553272** **1.00418478793884**

Low-Sociable **0.4743043** **0.970127947143046** **1.00668416106486**

;

**RUN**;

**PROC** **GENMOD** DATA=combined_data_across_tasks_2016;

MODEL SociabilityText = _NovelPreferred Gaze_AG_NS Look_AG_NS/ DIST=Binomial LINK=Logit type3;

**RUN**;

**PROC** **GENMOD** DATA=combined_data_across_tasks_2016;

MODEL SociabilityText = _NovelPreferred Gaze_AG_NS Look_AG_NS/ DIST=Binomial LINK=Logit type3;

**RUN**;

**PROC** **GENMOD** DATA=combined_data_across_tasks_2016;

MODEL SociabilityText = _NovelPreferred / DIST=Binomial LINK=Logit type3;

**RUN**;

**PROC** **GENMOD** DATA=combined_data_across_tasks_2016;

MODEL SociabilityText = Gaze_AG_NS / DIST=Binomial LINK=Logit type3;

**RUN**;

**PROC** **GENMOD** DATA=combined_data_across_tasks_2016;

MODEL SociabilityText = Look_AG_NS/ DIST=Binomial LINK=Logit type3;

**RUN**;
