# Supplementary figures and images for: Early Predictors of Impaired Social Functioning in Male Rhesus Macaques (Macaca mulatta)
Source: PLoS One. 2016 Oct 27;11(10):e0165401. doi: 10.1371/journal.pone.0165401 (PMC5082922; doi:10.1371/journal.pone.0165401)

25-16

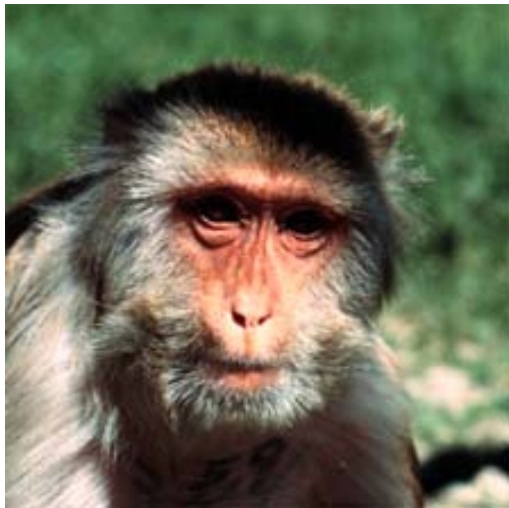

25-16

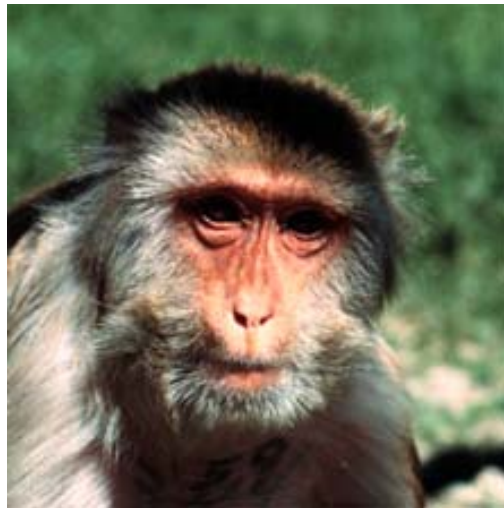

25-16

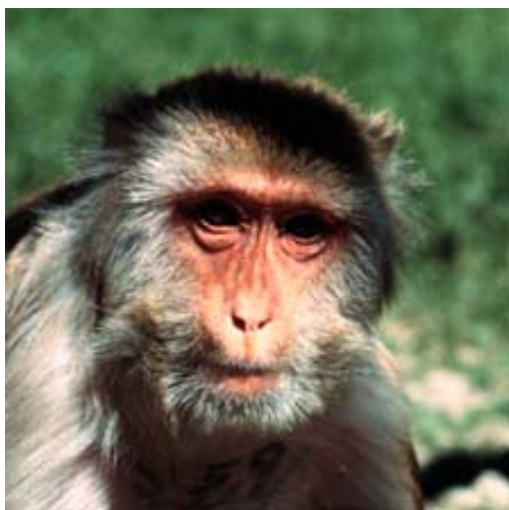

18-22

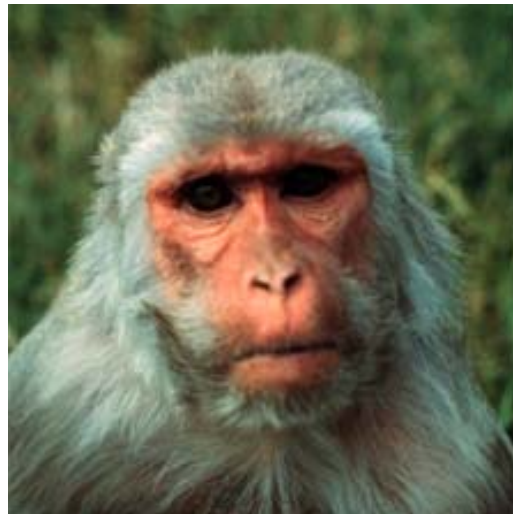

18-22

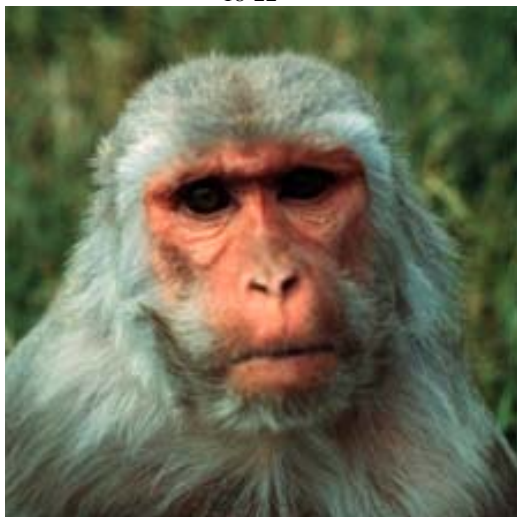

25-16

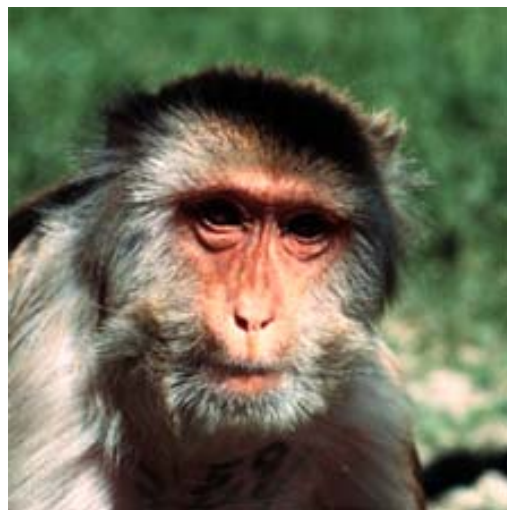

15-34

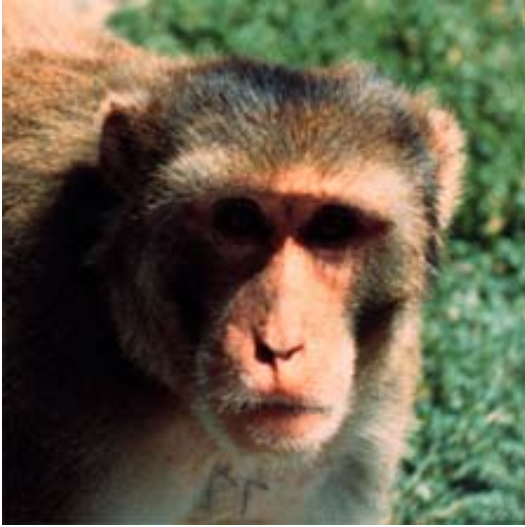

25-14

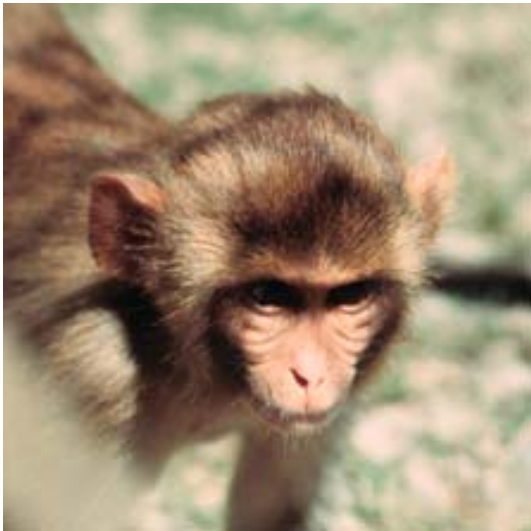

15-34

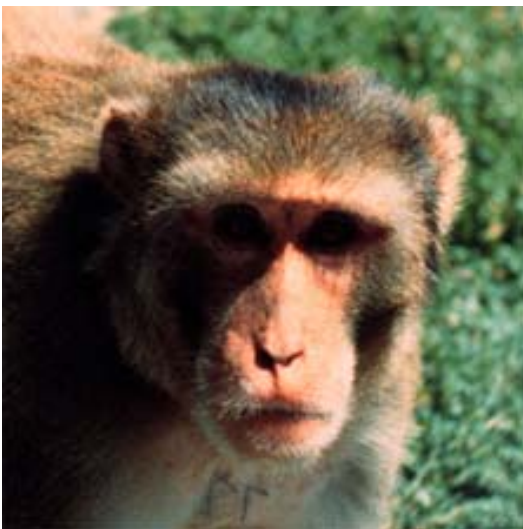

15-34

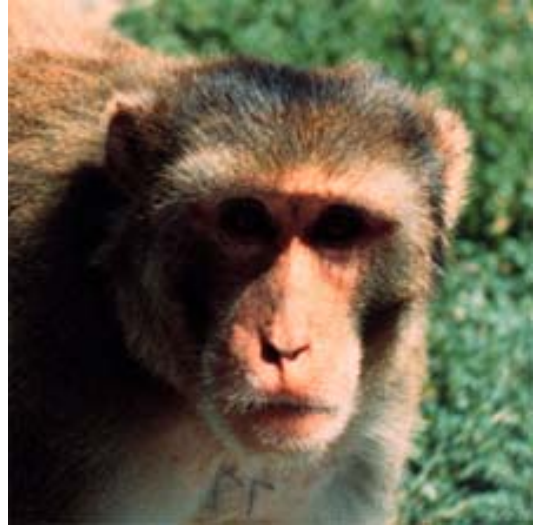

15-34

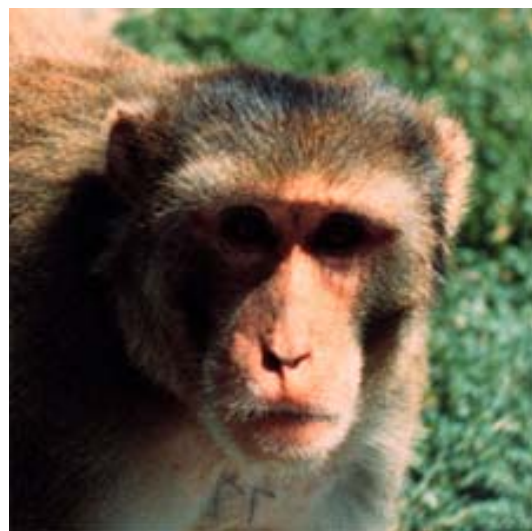

25-14

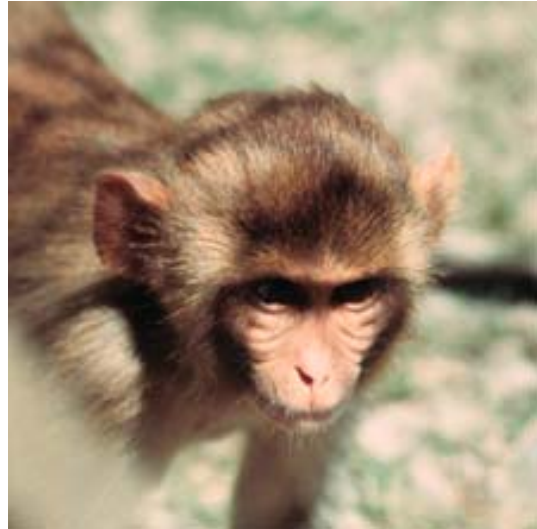

21-17

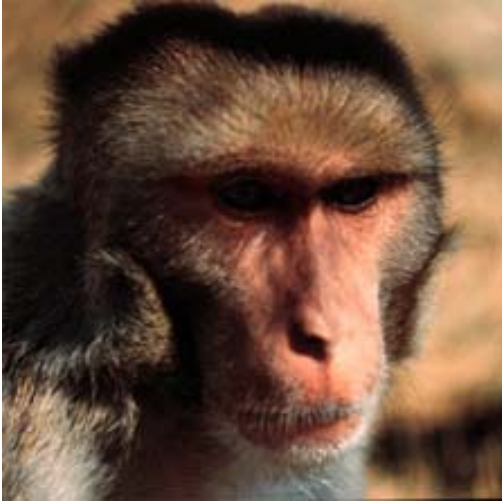

21-17

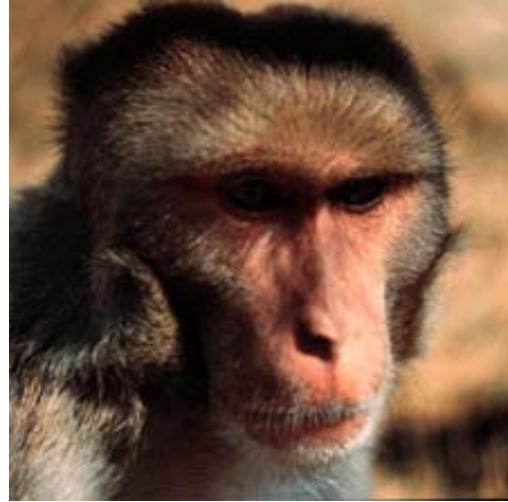

21-17

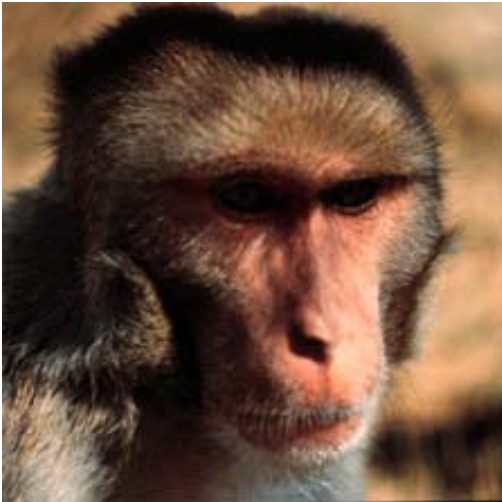

18-14

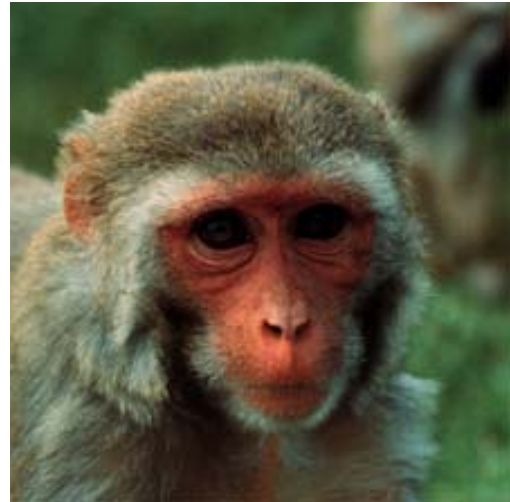

18-14

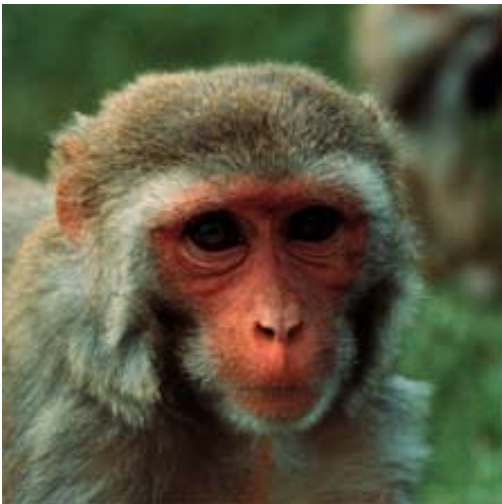

21-17

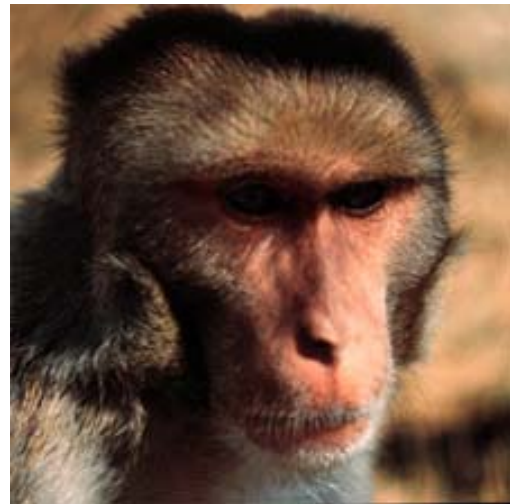

22-08

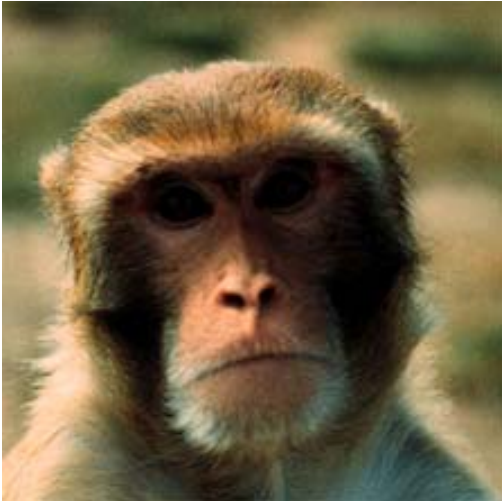

22-08

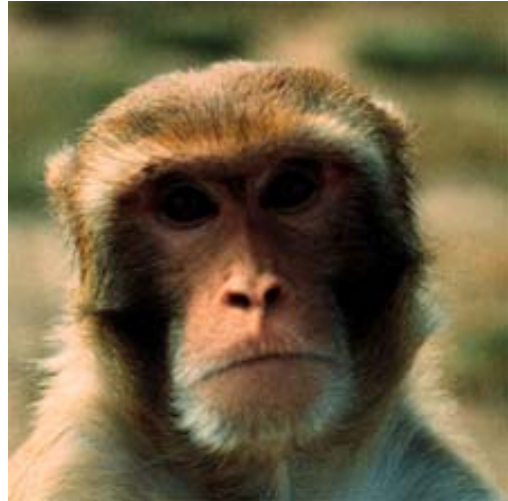

22-08

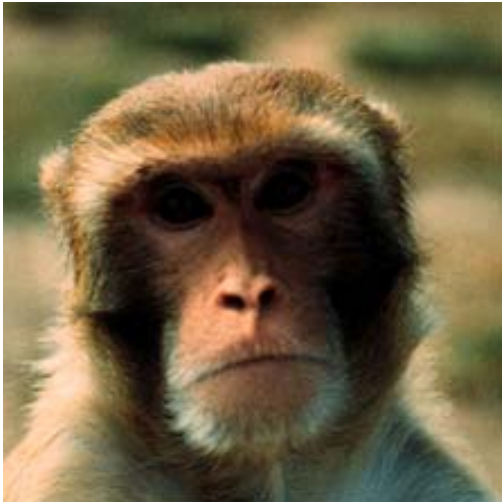

22-13

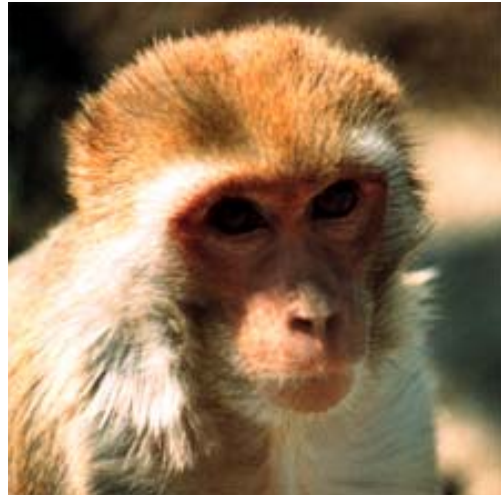

22-13

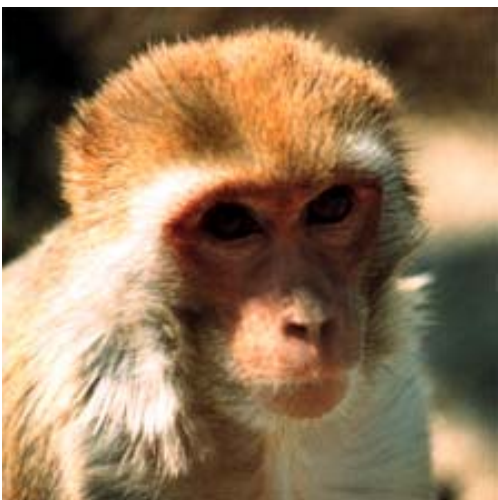

22-08

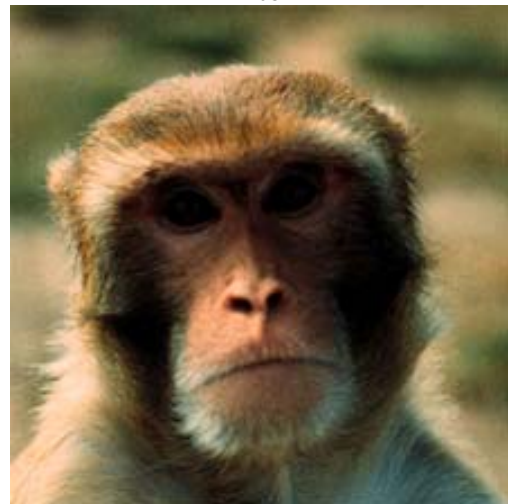

22-02

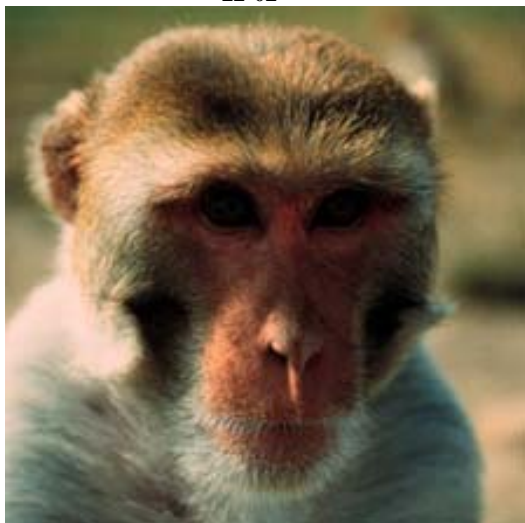

22-02

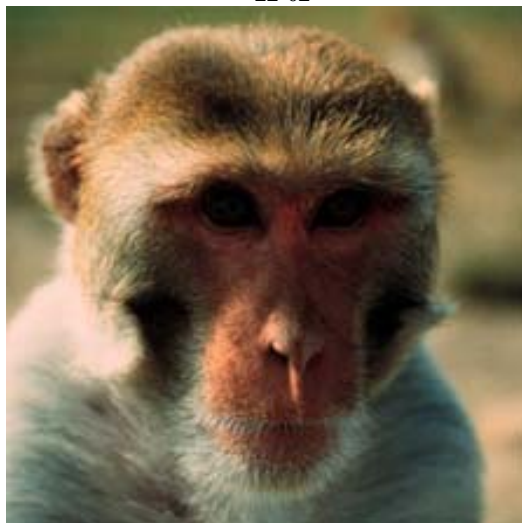

22-02

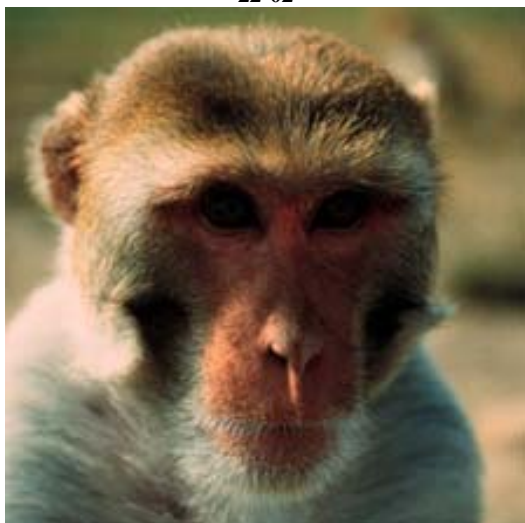

21-29

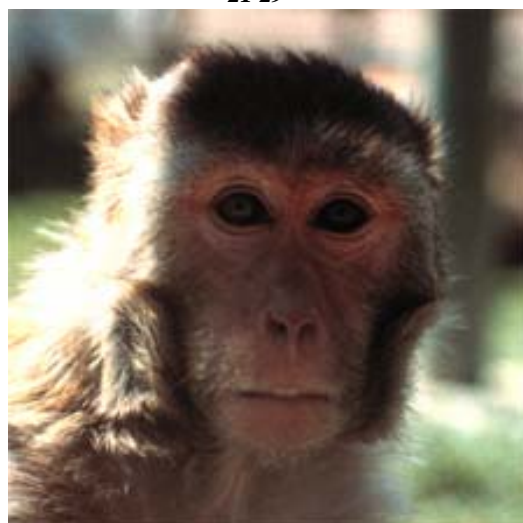

21-29

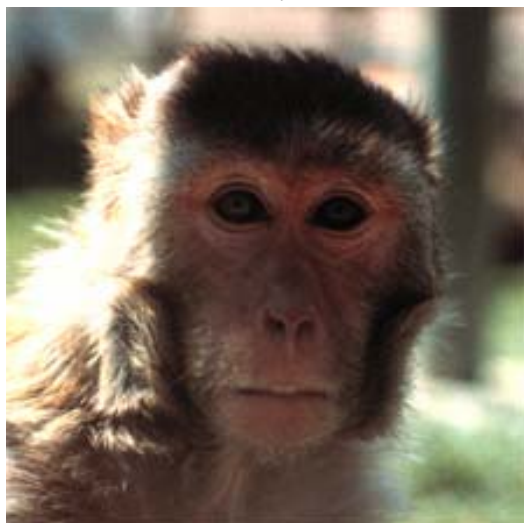

22-02

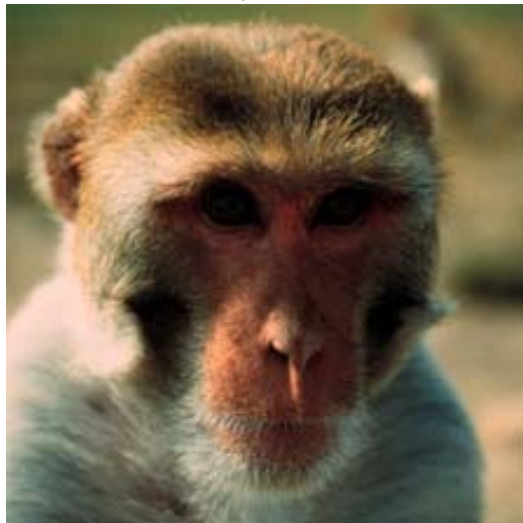

19-30

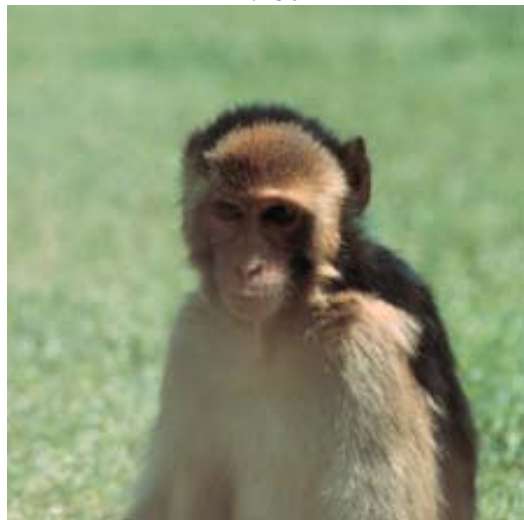

19-30

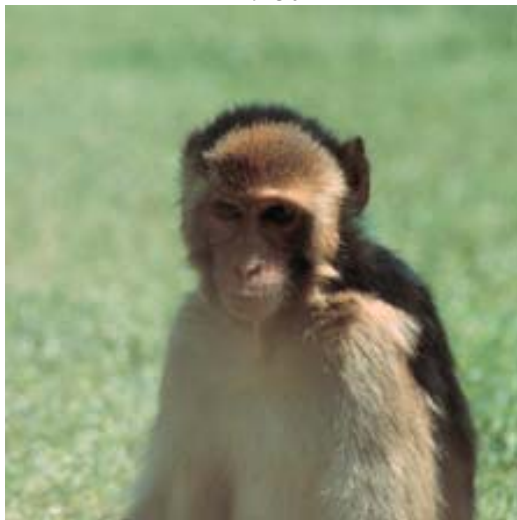

16-01

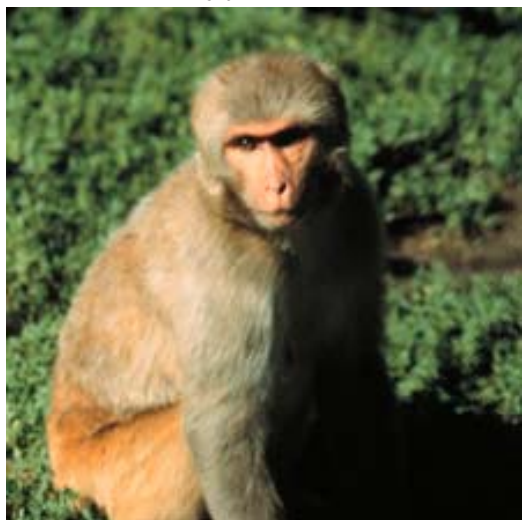

19-30

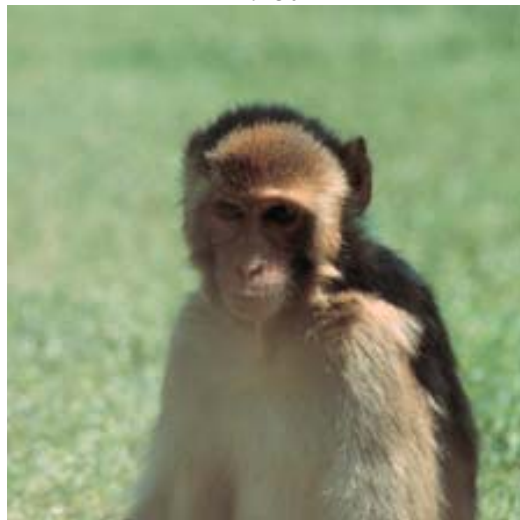

19-30

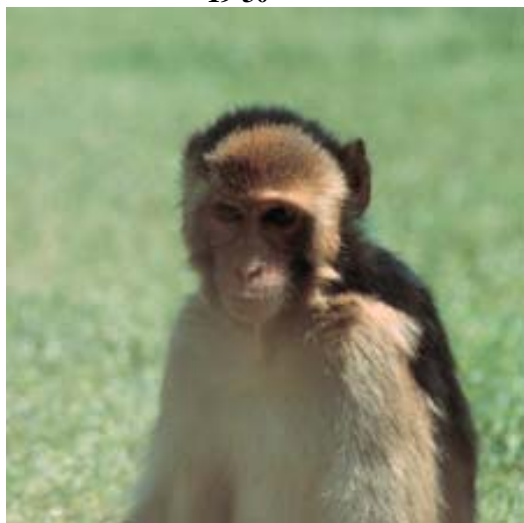

16-01

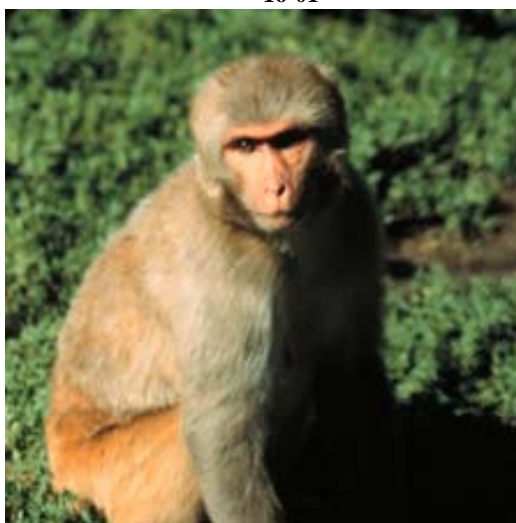

18-03

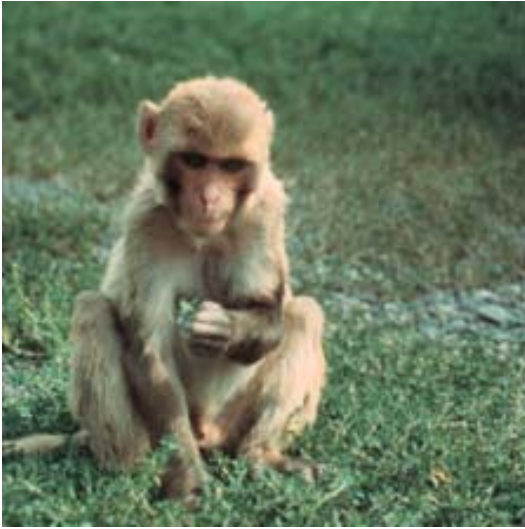

18-03

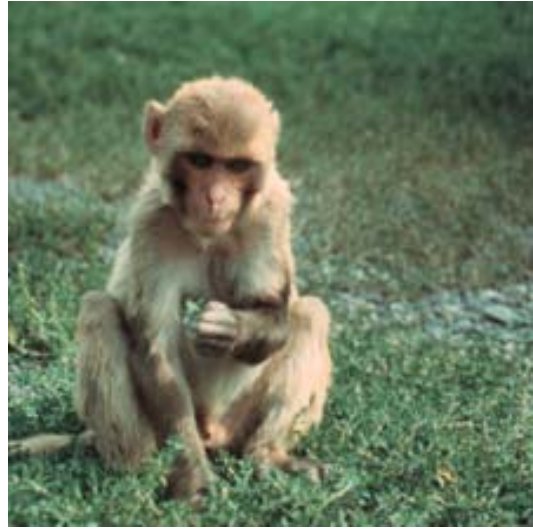

18-24

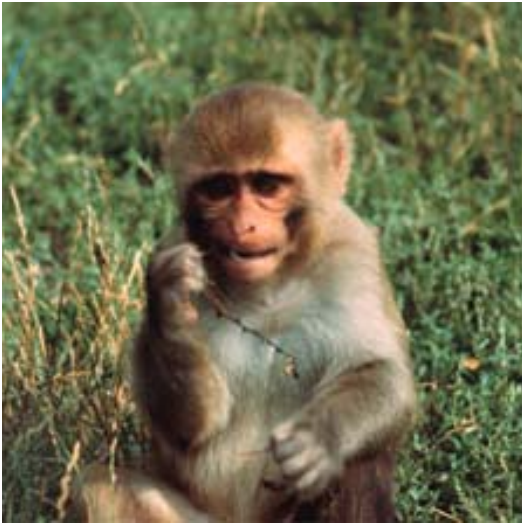

18-03

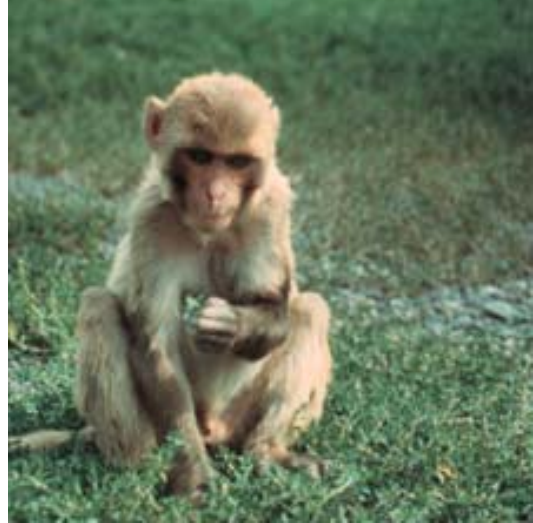

18-03

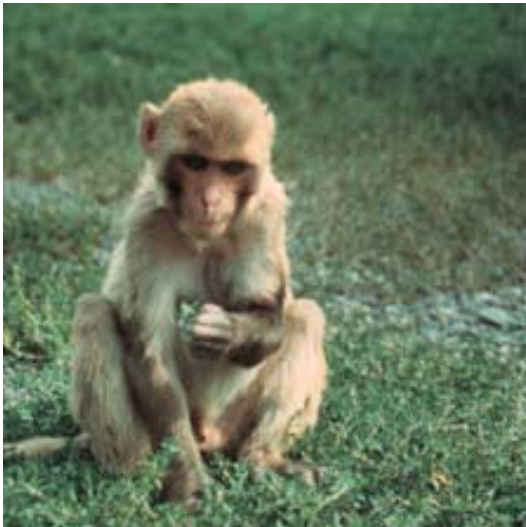

18-24

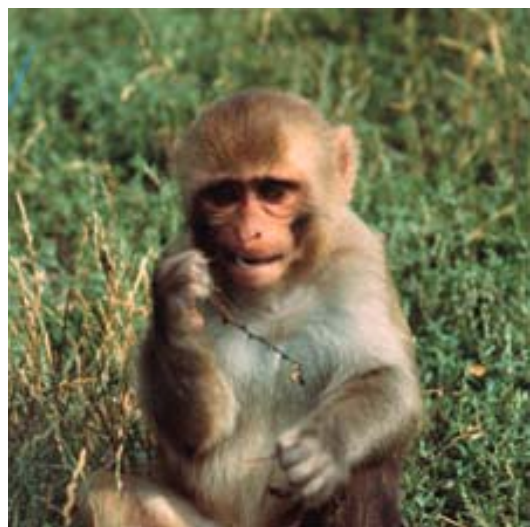

Supplement: S1 Fig — (PDF) [file pone.0165401.s002.pdf]
